# Subtelomere organization in the genome of the microsporidian *Encephalitozoon cuniculi*: patterns of repeated sequences and physicochemical signatures

**DOI:** 10.1186/s12864-015-1920-7

**Published:** 2016-01-07

**Authors:** Ndongo Dia, Laurence Lavie, Ngor Faye, Guy Méténier, Edouard Yeramian, Christophe Duroure, Bhen S. Toguebaye, Roger Frutos, Mbayame N. Niang, Christian P. Vivarès, Choukri Ben Mamoun, Emmanuel Cornillot

**Affiliations:** Unité de Virologie Médicale, Institut Pasteur de Dakar, 36 Avenue Pasteur, B.P. 220 Dakar, Sénégal; Clermont Université, Université Blaise Pascal, Laboratoire Microorganismes, Génome et Environnement, UMR 6023, CNRS, 63177 Aubière, France; Laboratoire de Parasitologie Générale, Département de Biologie Animale, Faculté des Sciences et Technologies, Université Cheikh Anta Diop, Dakar, Sénégal; Unité de Bioinformatique Structurale, UMR 3528 CNRS, Institut Pasteur, 25-28, rue du Dr Roux, 75015 Paris, France; Laboratoire de Météorologie Physique, OPGC UMR 6016 CNRS-Université Blaise Pascal, 24 Avenue des Landais, 63177 Aubière Cedex, France; CIRAD, UMR 17, Cirad-Ird, TA-A17/G, Campus International de Baillarguet, 34398 Montpellier, France; Section of Infectious Disease and Department of Microbial Pathogenesis, Winchester Building WWW403D, Yale School of Medicine, 15 York St., New Haven, CT 06520 USA; Institut de Recherche en Cancérologie de Montpellier, IRCM - INSERM U1194 & Université de Montpellier & ICM, Institut régional du Cancer Montpellier, Campus Val d’Aurelle, 34298 Montpellier cedex 5, France; Institut de Biologie Computationnelle, IBC, Campus Saint Priest, 34090 Montpellier, France

**Keywords:** Microsporidia, *Encephalitozoon cuniculi*, Subtelomere, Chromosome ends, Recombination, Multigene family

## Abstract

**Background:**

The microsporidian *Encephalitozoon cuniculi* is an obligate intracellular eukaryotic pathogen with a small nuclear genome (2.9 Mbp) consisting of 11 chromosomes. Although each chromosome end is known to contain a single rDNA unit, the incomplete assembly of subtelomeric regions following sequencing of the genome identified only 3 of the 22 expected rDNA units. While chromosome end assembly remains a difficult process in most eukaryotic genomes, it is of significant importance for pathogens because these regions encode factors important for virulence and host evasion.

**Results:**

Here we report the first complete assembly of *E. cuniculi* chromosome ends, and describe a novel mosaic structure of segmental duplications (EXT repeats) in these regions. EXT repeats range in size between 3.5 and 23.8 kbp and contain four multigene families encoding membrane associated proteins. Twenty-one recombination sites were identified in the sub-terminal region of *E. cuniculi* chromosomes. Our analysis suggests that these sites contribute to the diversity of chromosome ends organization through Double Strand Break repair mechanisms. The region containing EXT repeats at chromosome extremities can be differentiated based on gene composition, GC content, recombination sites density and chromosome landscape.

**Conclusion:**

Together this study provides the complete structure of the chromosome ends of *E. cuniculi* GB-M1, and identifies important factors, which could play a major role in parasite diversity and host-parasite interactions. Comparison with other eukaryotic genomes suggests that terminal regions could be distinguished precisely based on gene content, genetic instability and base composition biais. The diversity of processes assciated with chromosome extremities and their biological consequences, as they are presented in the present study, emphasize the fact that great effort will be necessary in the future to characterize more carefully these regions during whole genome sequencing efforts.

**Electronic supplementary material:**

The online version of this article (doi:10.1186/s12864-015-1920-7) contains supplementary material, which is available to authorized users.

## Background

*Encephalitozoon cuniculi* is a member of the phylum Microsporidia which are obligate intracellular parasites related to fungi [1] and which infect most animal taxa and some protists. The environmentally resistant spores of *E. cuniculi* can infect a wide range of mammalian hosts, and is recognized as an opportunistic human pathogen in immunocompromised patients [1, 2]. *E. cuniculi* is often described as a model organism for highly compacted genomes [3]. The high degree of host dependence leads an extreme reduction in the number of genes encoded in its genome [4].

The ~ 2.9 Mbp nuclear genome of *E. cuniculi* consists of 11 chromosomes ranging in size between 217 and 315 kbp [5]. Restriction mapping indicated that the ends of each chromosome share a common domain of ~15 kbp marked by the presence of one 16S-23S rDNA transcription unit [6]. Three *E. cuniculi* strains (I, II and III) have so far been identified on the basis of their immunological profile as well as by molecular analyses based on the presence of a variable number of GTTT repeats within the unique rDNA internal transcribed spacer separating the two rRNA-coding regions [7]. Comparative analyses of isolates differing in host and/or geographic origin revealed inter- and intra-strain karyotype variability [8, 9]. Chromosomal length polymorphisms (CLPs) were found to be in the form of insertion-deletion events (Indels) over 3–10 kbp in size, occurring within transition zones between rDNA units and chromosome cores [10].

The genome sequence of the *E. cuniculi* GB-M1 strain I which was reported in 2001 [4] was obtained using Whole Genome Sequencing (WGS). This effort revealed an unusually small genome containing about 2000 candidate protein-coding genes that are densely packed and very rarely interrupted by introns of short sequences. Although single-copy genes are predominant in this genome, the two ends of the smallest chromosome (chromosome I) share a common segment of ~ 37 kbp that encompasses the subtelomeric rDNA unit and covers a cluster of six genes [11]. Genome mapping have indicated that the chromosomes of *E. cuniculi* contains one rDNA gene at each extremity [6]. However due to an incomplete assembly of the genome of this parasite, only three rDNA units have so far been assembled. Chromosome ends assembly is inherently difficult and the process can be laborious even for reference genomes such as those of yeast, human or malaria parasites [12–16]. In many cases, sequencing of chromosome ends is performed independently using a combination of molecular and bioinformatics methods [17–22]. Full assembly of chromosome ends in yeast and humans using this approach revealed a mosaic of specific interchromosomal segmental duplications [14, 23, 24]. In human pathogens, repeated sequences in these regions encode factors involved in host-cell interaction and/or immune escape mechanisms [25–31].

Here we describe the final assembly of chromosome ends of *E. cuniculi* GB-M1 isolate. These ends represent about 15 % of the haploid size of the genome. The sub-terminal region of each chromosome is composed of large repeat units encoding components of four multigene families. These genes are organized in a novel mosaic structure of segmental duplications (EXT repeats) ranging in size between 3.5 and 23.8 kbp. We have mapped specific recombination sites at the boundary of the subtelomeric and coding-core regions of the chromosomes and propose that Double Strand Break Repair mechanisms are responsible for their mosaic organization. These genetic rearrangments may have play a major role in parasite diversity, virulence and host-pathogen interactions.

## Results

### Mosaic organisation of chromosome ends

The two ends of each chromosome (Σ) of *E. cuniculi* were designated as Σα and Σβ, referring to the 5’ and 3’ ends of the Watson strand of the sequence deposited in the database, respectively. Sequence comparison between the different chromosome ends was first performed with Miropeat software to identify gap free repeats (Additional file 1: Figures S1 and S2A) and confirmed by BLAST as previously described [32]. Our analysis revealed that the available sequence of the subtelomeric regions is incomplete for the following three reasons: (i) the rDNA-containing repeat is only found on both ends of chromosome I and on IVα extremity whereas previously reported physical mapping data indicated that each subtelomere includes the rDNA unit [6]; (ii) no genes belonging to gene families known to associate with chromosome ends were detected on IIIβ and IXα, and (iii) short duplicated sequences at far extremities are due to artificial truncations of subtelomeric specific repeats (Additional file 1: Figure S2B).

**Fig. 1:**
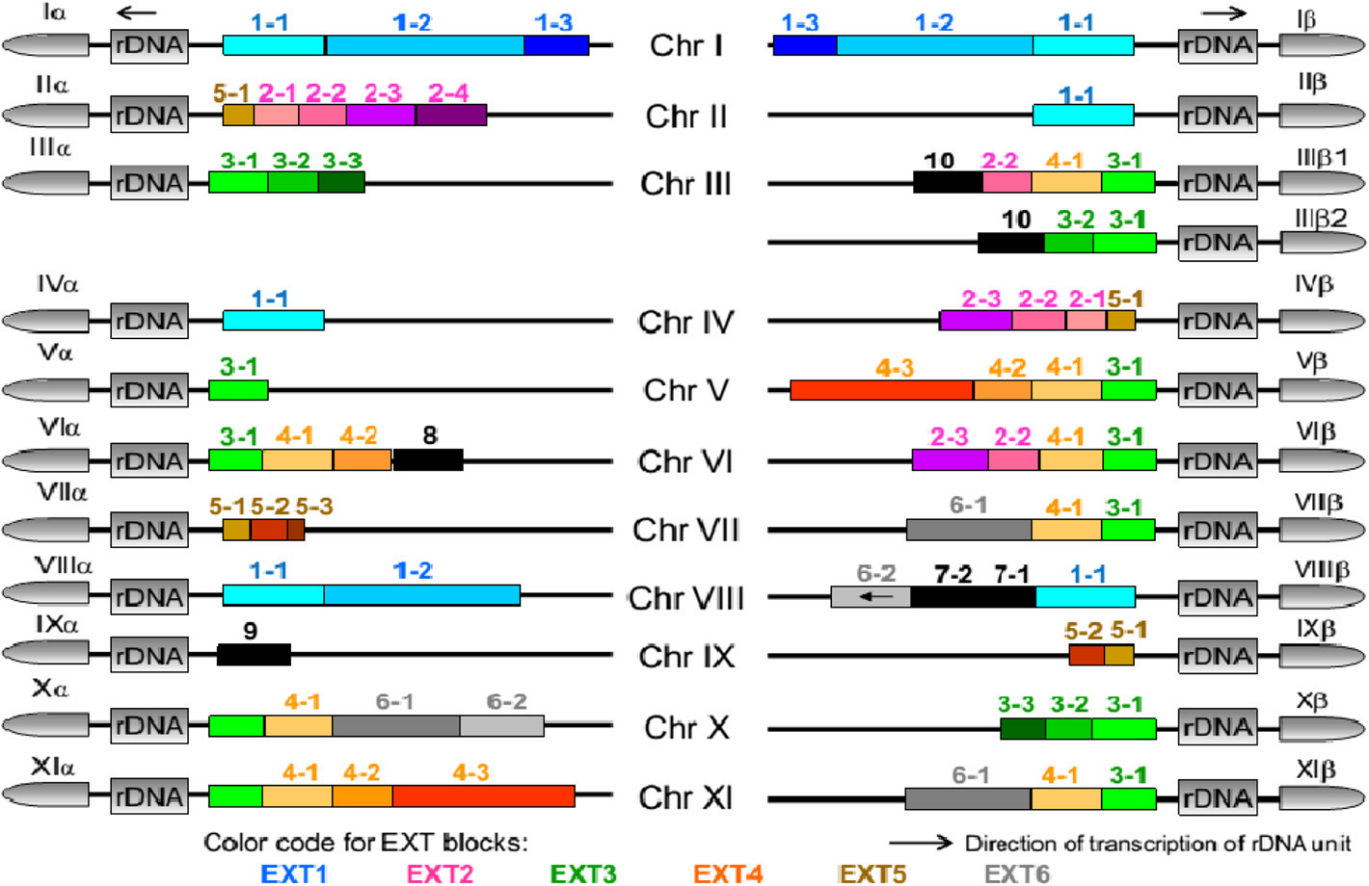
Mosaic organization of subtelomeric sequences in the genome of *Encephalitozoon cuniculi* GB-M1 (strain I). Numbering from I to XI refers to the haploid set of chromosomes. The α end of each chromosome is represented on the left and β end on the right. As the diploid genome of *E. cuniculi* GB-M1 displays two homologous chromosomes III having diverged at the β end, a total of 23 subtelomeres are described. Colour gradation was used to represent the different EXT sub-sequences issuing from the same EXT repeat. The scale has been conserved to represent the different EXT sub-sequences

To determine chromosome ends organization in *E. cuniculi* in GB-M1 isolate, long PCR reactions were performed to amplify the chromosome extremities between the rDNA region and the sequences available in the Genbank database (Additional file 1: Figure S3). Overall, we obtained about 26 kbp of new sequence data, which are accessible under accession numbers HG380753, HG967526, HG967527 and HG967528. The genome of *E. cuniculi* is diploid and the GB-M1 genome contains two chromosome III homologues differing by a single indel [6]. We isolated and sequenced the two different chromosome IIIβ extremities from PCR products (Additional file 1: Figure S4). Sequencing effort further revealed uncharacterized sequences at chromosome IIα and IXα extremities. The new DNA sequence characterized on chromosome IIα was also found to be present at chromosome IVβ extremity. The other extremities showed sequences in agreement with the data published so far. We deduce the sequence organisation between the end of available contigs and the rDNA region on the basis of PCR and sequencing. This first step was essential to establishing the existence of large blocks of adjacent repetition units clustered at the ends of *E. cuniculi* chromosomes. Using this approach, we defined 10 EXT repeats that are completely or partially repeated among the 22 chromosome ends (Fig. 1). They encode several CDS, including house-keeping genes (Additional file 1: Figure S5). Altogether, these data indicate that EXT repeats are components of the subtelomere in *E. cuniculi*.

**Fig. 2:**
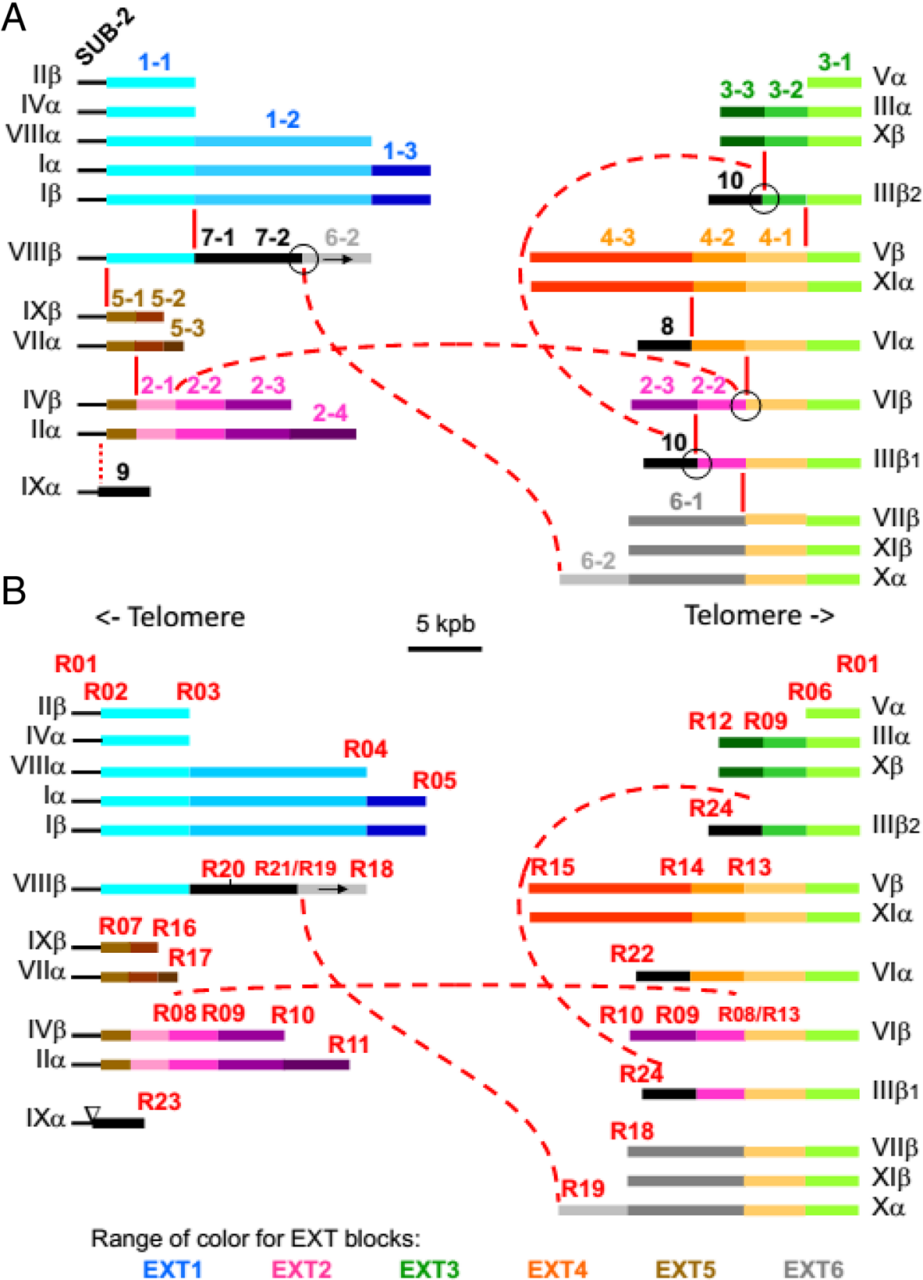
Distribution of EXT sub-blocks illustrating extensive variations of chromosome ends organisation. **a**. The assignation to two main types of EXT organization was performed on the basis of an anchoring with either recombination site R01 (on the right) or R02 (on the left) to the conserved SUB region. Breaks within EXT blocks are indicated by vertical red bars. Fusion events are marked by circles. The discontinuous lines connect the two recombination sites involved in the fusion. The inverted copy of EXT6-2 is shown by an arrow.The inverted triangle indicates that EXT9 contains divergent SUB-2 and EXT1-1 sequence presenting large deletions. Colour gradation was used to represent the different EXT sub-sequences issuing from a same EXT repeat. The scale has been conserved to represent the different EXT sub-sequences. **b**. Distribution of the recombination sites (R) delimiting EXT sub-blocks. These sites are often hot spots of sequence rearrangement and were identified by their telomere-associated distal sequence which was conserved after genetic events. The two IIIβ variants originate from a homologous recombination event that involving two R09 sites, that was conservative of the proximal block (EXT10). The distal sequence of these sites is highly conserved over about 1000 bp (98 % identity without gap) between EXT2-2 (R09a) and EXT3-2 (R09b)

*E. cuniculi* subtelomeric region includes the rDNA unit and surrounding DNA with a maximum length of 16 kbp on chromosome I. This so-called SUB region is present and identical at all extremities (Fig. 1). It has no similarity with either the EXT repeats defined above or with the core sequences in the *E. cuniculi* genome even at CDS level. This region lacks the stretches of repetitive sequences characteristic of the telomeric ends. Based on PFGE data [6], the missing data between the SUB region and the telomere could be estimated to about 4 to 5 kpb. The SUB region contains a small TAAA microsatellite, upstream of the rDNA unit, at position 11800 on Iα. A small indel between chromosome I and chromosome IV (1 bp in a C/G stretch at position 10578 on Iα) is the only polymorphism found between the SUB copies in the *E. cuniculi* GB-M1 genome. The length of the SUB region is variable near the EXT repeats (Figs. 1 and 2a). The first breakpoint at 5.3 kbp from the beginning of the rDNA unit was labelled R01 (recombination site 01) and was present on IIIα, Vα and Xβ ends (Fig. 2b). It marks the transition between the SUB region and the EXT3 repeat. The second breakpoint (R02) at 7.3 kbp from the beginning of the rDNA unit indicates the transition between SUB and either EXT1 (found on Iα, Iβ, IIβ, IVα and VIIIβ) or EXT5 (found on VIIα and IXβ, Fig. 2b). The region between R01 and R02 sites, called SUB-2, is found on half of chromosome ends (Fig. 2a).

### Chromosome ends assembly

All *E. cuniculi* GB-M1 chromosomes contain a 16 kbp large inverted repeat at each extremity (the SUB region). In some cases, the EXT organization increases the size of the inverted repeat at chromosome extremities. The chromosomes with large symmetrical organisation are chromosome I (entire EXT1 repeat), VI (EXT3-1 + EXT4-1) and VIII (EXT1-1 sub-sequence). *Consequently, the E. cuniculi* chromosomes are the largest amphimeric molecules described so far (Fig. 1). Each EXT repeat is generally present once per extremity. We identified sub-sequences because some EXT copies were truncated. Breakpoints within EXT are scattered among the 22 extremities (Figs. 1 and 2). No physical link was established between the EXT2 repeat and the SUB region in the first release of the *E. cuniculi* genome. Sequencing of Long-PCR products specific of extremities IIα and IVβ revealed that the SUB-to-EXT2 junction occurs via a SUB/EXT5 sequence organization (Fig. 1).

EXT7 and EXT8 are unique in the genome sequence of *E. cuniculi* GB-M1 strain, but are of EXT origin. EXT8 was characterized on the basis of BLAST homologies shared with other EXTs, particulary with regard to CDSs ECU06_0090 and ECU06_0100. Sequence of IXα extremity shows the presence of a degenerated copy of EXT5. The sequence is marked by a large deletion in the EXT1-1 region (Additional file 1: Figure S5E). We therefore postulate that the IXα extremity represent an early step in the evolution of a new EXT (EXT9).

**Fig. 3:**
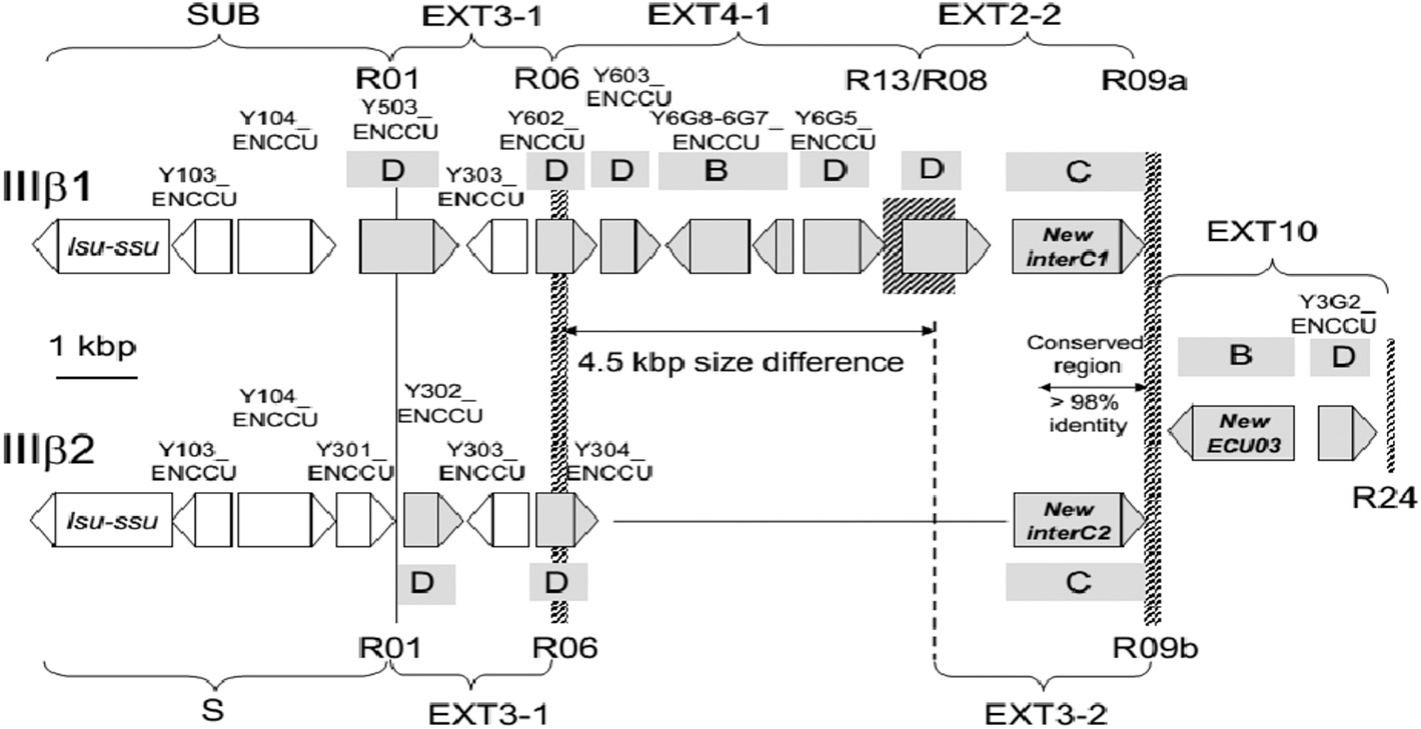
Sequence features justifying Chromosome Length Polymorphism (CLP) for two chromosome III homologues in *E. cuniculi* GB-M1. CDSs are represented by boxes with arrows indicating transcription direction and are ordered from telomere (left) to chromosome core (right). Grey colour is applied to the members of three multigenic families: *interB*, *interC* or *interD*. Name and/or Uniprot accession number are given for CDSs characterized by the genome project. The telomere-associated distal parts of the two extremities are identical up to R06 recombination site. The region of homology between the two extremities starts again just upstream of EXT10. In between, the EXT4-1-EXT2-2 organisation is 4.5 kbp longer than EXT3-2. Vertical bars show recombination sites. Their width covers the region where they are expected to be located. R01 is the only site that is precisely mapped in this region. The IIIβ1 and IIIβ2 EXT10 copies are differing by the number of copies of a CATCA microsatellite extending 3’ of *NewECU03* at ~ 50 bp of R09. Very few mismatches are observed between EXT2-2 and EXT3-2 over ~ 1000 bp at the left-hand part of R09. The IIIβ2 sequence is strictly identical to VIβ end up to R09 but significant homologies persist downstream of R09 up to the middle of the *NewECU03* gene. Both EXT2-2 and EXT3-2 display a G indel in a polyG repeat restoring a complete *interC* CDS (*interC1* and *interC2* genes are divergent in 5’)

Characterization of the two IIIβ extremities (IIIβ1 and IIIβ2) was particularly difficult to achieve (Fig. 3). This organisation was determined based on long-read assembly from subclones obtained from a long-PCR product and it was confirmed using data from the genome project. The IIIβ1 consensus shows a genomic organization that is close to the VIβ extremity with repeats order SUB/EXT3-1/EXT4-1/EXT2-2. The EXT-to-core transition is made by a new EXT block (EXT10) encompassing the *NewECU03* gene characterized at transcriptional level by Dia et al. [33]. PCR amplifications, sequencing and reassembly of reads from the genome project have shown that the EXT10 and EXT-to-core transition are conserved among the two IIIβ chromosome copies (Figs. 1 and 3, Additional file 1: Figure S4). The IIIβ2 organization is highly homologous to IIIα providing a new example of large amphimeric molecule (SUB/EXT3-1/EXT3-2). High sequence similarities between EXT2-2 and EXT3-2 may explain the difficulties in discriminating between the two IIIβ extremities in the previous genome assembly. The size difference between the two IIIβ genomic organisations reach 4.5 kbp which is consistent with the PFGE-estimated size polymorphism [6]. The present study revealed that the size polymorphism between chromosome III homologues results from an unequal exchange of chromosome ends. This exchange likely resulted from a homologous recombination in the highly conserved sequence between EXT2-2 and EXT3-2.

### Recombination sites border EXT repeats

The irregular distribution of subtelomeric repeats among the different chromosomes of *E. cuniculi* GB-M1 indicates a significant reshuffling of chromosome ends (Fig. 2a). A striking feature of this organisation is the gradient of diversity in EXT composition from the rDNA unit to the coding core of the chromosomes (Fig. 1 and Additional file 1: Figure S6). A total of 24 recombination sites (R01 to R24) were mapped and identified on the basis of their distal sequence (Fig. 2b). Some of them are involved in more than one transition event in the *E. cuniculi* GB-M1 genome and might be considered as hotspots of sequence rearrangement (R02, R03, R05, R06, R08, R10, R12, R13, R15, R18 and R19, Fig. 2b and Additional file 1: Figure S6). R01, R02, R18 and R19 were the only sites that could be precisely mapped. In this study, the other recombination sites could only be approximately mapped (average resolution of 100 bp, Additional file 1: Figure S5). The R01 recombination site is located in the subtelomere and is associated to the SUB-EXT3 transition. Fifteen recombination sites are associated with the EXT-to-core transitions. R03, R05, R10, R12, R15 and R18 are associated with two EXT-to-core transitions. The recombination sites R03, R04, R06, R10, R16 and R18 result from EXT truncations. The R24 site is present twice in the genome and at a different distance from the R01 site because of the presence of two IIIβ EXT sequence organisations (Fig. 3 and Additional file 1: Figure S6).

Nine recombination sites (R02, R03, R06, R07, R08, R09, R13, R14 and R21) are associated with internal EXT rearrangements responsible for various arrangements of EXT repeats at the different chromosome extremities (Figs. 1 and 2). R06 recombination site is at EXT3-EXT4 transition; the R07 site is associated with the EXT5-EXT2 transition; the R13 recombination hot spot is responsible for a rare translocation event at VIβ and IIIβ1 extremities. Consequently, the R09 site is located in two different EXT regions (EXT2-2 and EXT3-2) that share strong homologies in their proximal extremities (Fig. 3). This site is in the area of the transition with EXT10 both at IIIβ1 and IIIβ2 extremities. R13 site is also at the EXT4-EXT6 transition. The R14 site corresponds to the EXT4-EXT8 transition. The R21 recombination site is associated with the EXT6-2 inverted copy at EXT6-EXT7 transition. In this case R21 recombined with R19. According to Fig. 2, inversion of EXT repeats is a rare event in the genome of the *E. cuniculi* GB-M1 isolate.

### EXT repeats-associated multigene families

An important finding in this study is that EXT repeats differ in gene content, some of them encoding housekeeping genes. An aminopeptidase-dihydrofolate reductase (*dhfr*)-thymidylate synthase (*ts*) gene cluster is indeed specific to EXT1-2 (repeated three times in the genome). Three other adjacent genes encoding serine hydroxymethyltransferase, an ABC transporter and a protein of the NAP/SET family are located in the EXT1-3 sub-sequence. The EXT1-3 repeat is restricted to chromosome I. The sub-sequence EXT7-2 contains the ribosomal protein RL12-encoding gene. We estimate that ~ 150 CDS are associated with chromosome ends in the current assembly of the *E. cuniculi* GB-M1 genome.

**Fig. 4:**
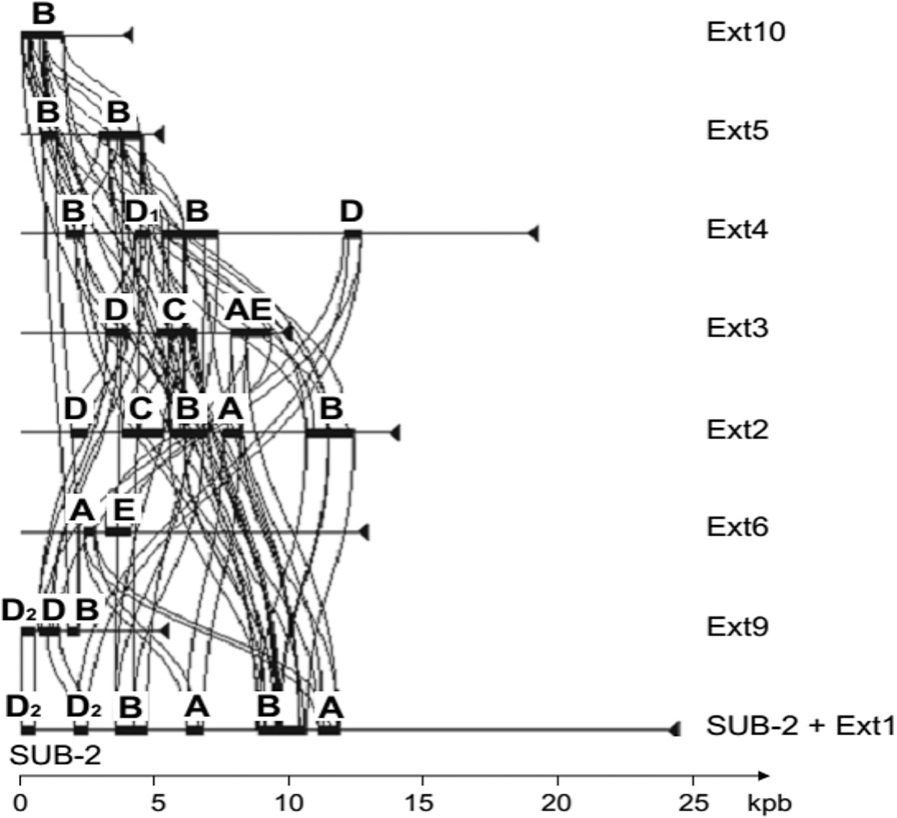
Miropeat comparison of eight *E. cuniculi* EXT blocks (threshold 250). The detected repeats correspond to CDSs classified into four distinct *inter* gene families (*interAE*, *interB*, *interC* and *interD*). EXT1 sequence was extracted from Iα end, EXT2 from IIα, EXT3 from Xβ, EXT4 from XIα, EXT5 from VIIα, EXT6 from Xα, EXT7 from VIIIβ and EXT8 from VIα (all these chromosome ends refer to Genoscope genome release). EXT9 was extracted from entry HG967528 (IXα new consensus) and EXT10 from entry HG967526 (IIIβ new consensus). EXT7 and EXT8 were too divergent from other EXT blocks to be presented on the diagram, at the used threshold value. The A and E repeats are located inside the same CDS that is therefore designated as *interAE*. The E repeat is more variable than A. Some similarity exists between InterAE and InterB proteins (PfamA domain DUF1609). Only two EXT blocks (EXT2 and EXT3) harbour C-type sequences but other copies of these sequences are found in the core regions of chromosomes IX, X and XI. One divergent *interC* copy has been identified in EXT7. As many recombination sites are mapped within *interD* genes, D repeat have been separated into two parts D1 and D2

Sequence comparison of EXT repeats revealed that they are composed of a common set of repeats called A, B, C, D and E (Fig. 4). All of these sequences overlap with predicted CDSs. The presence of four multigene families was confirmed by gene-to-gene comparison at the genomic scale. They were named *interAE*, *interB*, *interC* and *interD.* Repeats A and E refer to the same gene family. EXT3 and EXT6 encode the C- and N-terminal regions of a same putative proteins, respectively (Fig. 4). More divergent copies of *interAE* genes exist, the A repeat being the only conserved part of the gene (Fig. 4 and Additional file 1: Figure S5). Some *interAE* genes are pseudogenes because of the presence of a frameshift between the A and E repeats (e.g. EXT1-2) or due to the absence of the E corresponding sequence (e.g EXT2-3 at IVβ extremity, Additional file 1: Figure S5). Multigene families interB, interC and interD also contain pseudogenes. Overall, we found 19 *interAE*, 30 *interB*, 23 *interC* and 78 *interD* related CDS in the *E. cuniculi* genome (details in Additional file 1: Figure S5).

The Pfam database identified four protein domains of unknown function among the proteins encoded by these genes: 1) DUF1609 domain was identified because of the high level of sequence similarities that exist at the amino acid level between the C-terminus part of InterAE (encoded by inter-regions A of *interAE* genes) and InterB proteins; 2) DUF3654 domain corresponds to the N-terminal part of the proteins encoded by full *interAE* and *interB* genes; 3) DUF1686 is found in *interC* genes encoded polypeptides; and 4) DUF2463 is found in InterD proteins. More divergent sequences were found in the Uncharacterized Protein Family entries of UNIPROT Knowledgebase: UPF0328 (*interD*), UPF0329 (*interAE* and *interB*). These domains were also found, albeit less frequently, in telomere-associated ORFs in other *Encephalitozoon* species [34, 35]. The *interB* gene family is also present in more distant microsporidian species *Vittaforma cornea* and *Anncaliia* (*Brachiola*) *algerae* [33].

While *interAE, interB* and *interD* families are restricted to subtelomeres, twelve additional *interC* genes were found on three chromosome cores. Eight genes are found on chromosome IX and two on chromosomes X and XI. The orientation of all *interAE* and *interB* genes is the same and the genes are transcribed in the same orientation as the rDNA unit, i.e. towards the telomere. The *interC* and *interD* genes are transcribed in the opposite direction, i.e. in a centripetal way. Experimental evidence for transcription capacity has been previously shown for *interB* genes [33]. Most of the recombination sites are associated with these multigene families. A large part of the polymorphism in *interAE* gene family is due to the presence of the R03, R10 and R12 recombination sites within the E inter-region (Additional file 1: Figure S5A-C). Rearrangement events between *interB* genes are less common. Some of these genes are disrupted by the R07 site (Additional file 1: Figure S5B and E) and two recombination sites are associated with *interB* 5-prime sequences (R14 and R17, Additional file 1: Figure S5D and E). The duplicated R09 recombination site and R20 site are adjacent to the 3-prime region of *interC* genes (Additional file 1: Figure S5 B, C and G).

Six breakpoints (R01, R02, R06, R08, R13 and R16) are found within *interD* genes (Additional file 1: Figure S5A-F); with R02, R06, R08 and R13 as hot spots of recombination. Genes from the *interD* multigene family are the most overhauled compared to the three other gene families. The *interD* consensus gene could be divided into two segments, D1 and D2, encoding the respective N-terminal and C-terminal protein regions (Fig. 4). The D1 part of the genes extends over the promoter region. Most *interD* genes encode a full protein of about 260 residues. Some recombination events results in the linkage of an *interD* segment (D2) to a non-specific ORF (e.g. at R01 recombination site associated to the transition with EXT1-1 or EXT3-1, Additional file 1: Figure S5A). This tight relationship between multigene families and recombination sites support the model that all EXT repeats have a common origin and that chromosome end organisation results mostly from homologous recombination events.

### Nucleotide composition of chromosome ends

**Fig. 5:**
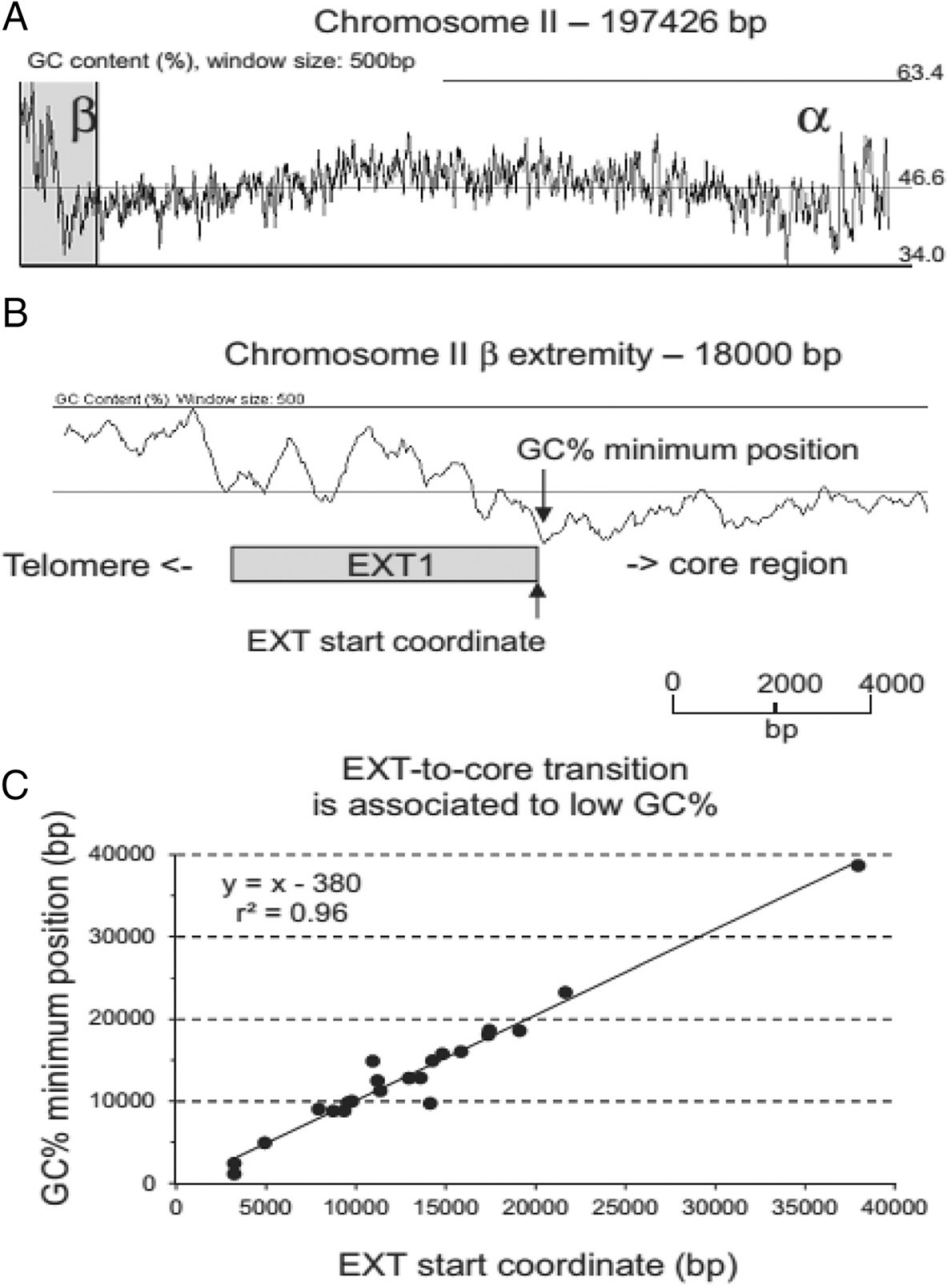
The EXT-to-core transition is associated to a shift of GC % average values. **a** The GC % is calculated over one horizon (windows size 500 bp) along the sequence of chromosome II. All chromosome extremities are characterised by a high GC content in *E. cuniculi*. The transition with the central part of the chromosome is improved by the bell-like shape of the GC % curve over the coding core region. The GC % values are high at the centre of the molecules. The GC content of the chromosome core sequence diminishes progressively from the centre to the end by accumulation of local minima. The chromosome II is represented in a opposite orientation. **b** EXT1 repeat in a GC rich region at IIβ extremity (shaded region in A). All EXT repeats (except EXT7) are associated with high GC % values. After touching its minimal value, the GC-curve is growing more or less rapidly depending on extremities when EXT sequences are read in a core to telomere orientation. The IIβ extremity is part of extremities presenting a slow growing curve at the EXT-to-core transition. **c** EXT-to-core transitions are associated to low GC %. The GC % minimum value was plotted in function of the EXT-to-core transition for each extremity. Coordinates were calculated from the corresponding end of the consensus sequence

The extremities of *E. cuniculi* chromosomes contain a high GC content (average at 60 %, maximum at 66 %) compared to the central parts of the molecules (Fig. 5a). The GC content is calculated using a sliding window whose order of magnitude is usually around 500 bp. This approach is useful to study long-distance tendency but is characterized by a low signal-to-noise ratio and low resolution (Fig. 5 and Additional file 1: Figure S7). As described by other authors [11, 36], all the *E. cuniculi* chromosome cores share a bell-shaped GC % curve with a nearly central apex reaching 54–59 % (up to 9 points above genome average). The sequences at the transition with EXT blocks exhibit the lowest values (24 % GC content on average, Fig. 5). The transition rate value between the minimum GC % value associated with a chromosome end and the maximum value observed in the nearby EXT region could increase sharply or slowly as shown on Additional file 1: Figure S7.

**Fig. 6:**
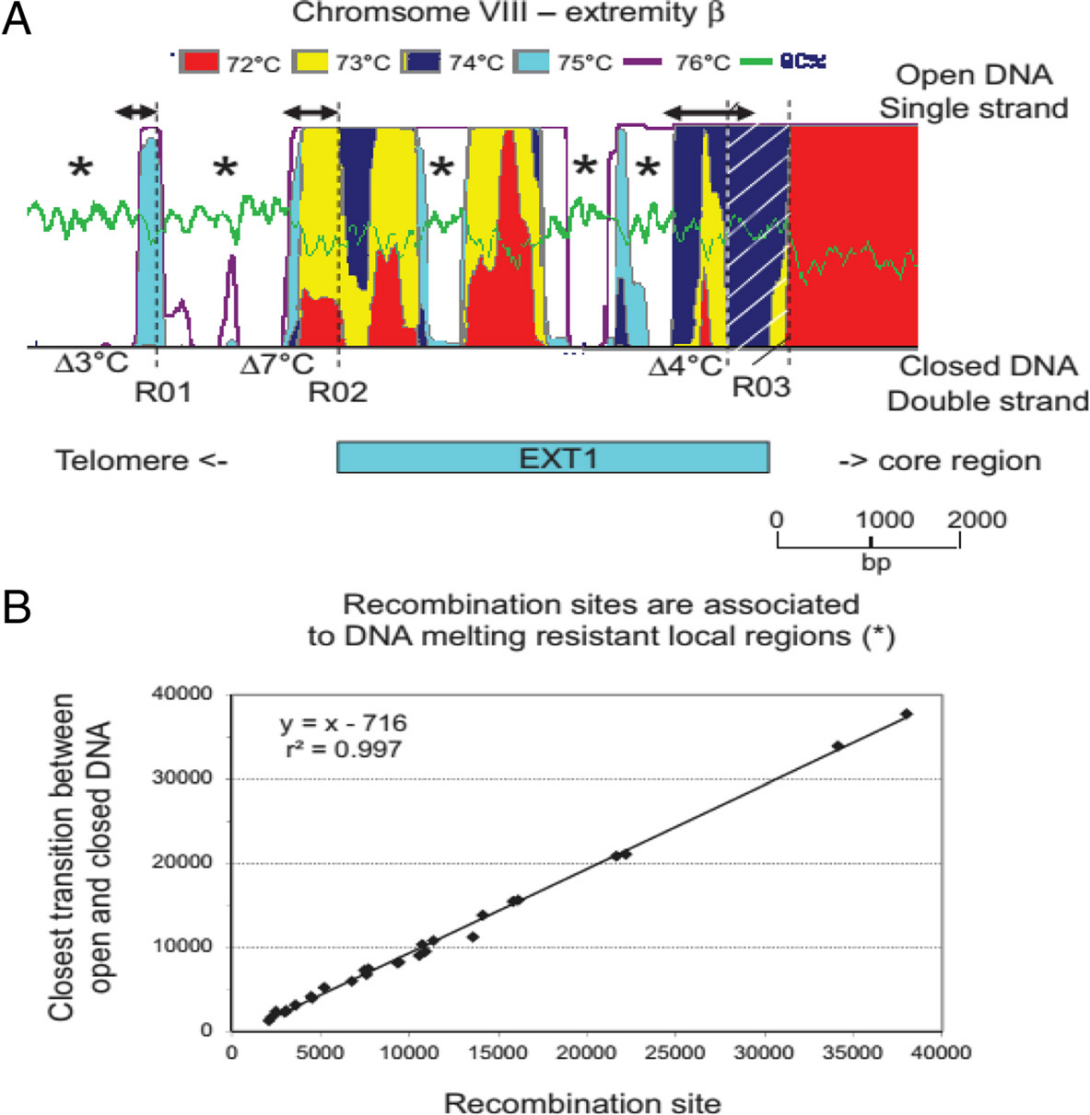
Recombination sites are located close to DNA melting resistant regions. **a** The state of the DNA molecule between “closed” (double stranded) and “open” (single stranded) was measured at different temperature along the VIIIβ extremity. The GeneFizz software was implementing an algorithm which was able to calculate melting state of the DNA molecule locally [37]. This algorithm had no mathematical horizon (a sliding window of 20 bp was used in the present study). Curves calculated at different temperature were superimposed to identify regions where the transition between closed and open state requires a temperature increase of several degrees (*). Doubled arrows at the top of schema indicate the distance between the recombination site and transition zone. Recombination sites have been mapped in centromere proximal part of a closed region. We never found recombination sites within a closed region, but the distance to a closed region vary from a hundred of base pair to one kbp. The hatched area associated to R03 recombination site covers the region where it is expected to be located. R01 and R02 have been precisely mapped. **b** Recombination sites are associated with DNA melting resistant regions. The transition between closed and open state of the DNA molecule was plotted in function of the position of the recombination site for all sites at all extremities. Coordinates were calculated from the corresponding end of the consensus sequence

Two alternatives approaches have been used to evaluate in more details the base composition at chromosome ends. We first used the GeneFizz software implementing a helix-coil transition model to search for local variations in DNA melting properties (i.e. the transition between the close and open state corresponding respectively to the double stranded and the single stranded form of the DNA molecule [37, 38]). The state of the DNA molecule was measured at different temperatures along the whole chromosome with a sliding window of 20 bp [37]. Curves calculated at different temperatures were superimposed to identify regions where the transition between closed and open states requires an increase of the temperature by several degrees (Fig. 6 and Additional file 1: Figure S8). The GC rich regions of the genome such as chromosome ends are in closed state at 74 or 75 °C. It was possible to identify rapid transition between open and closed states in those regions when temperature is increased because of the high resolution of the GeneFizz local analysis of the DNA melting properties. Recombination sites found in EXT repeats are associated with open-close transitions of more than 3 °C (Fig. 6b). The average distance between the recombination site and the temperature shift is approx. 700 bp.

**Fig. 7:**
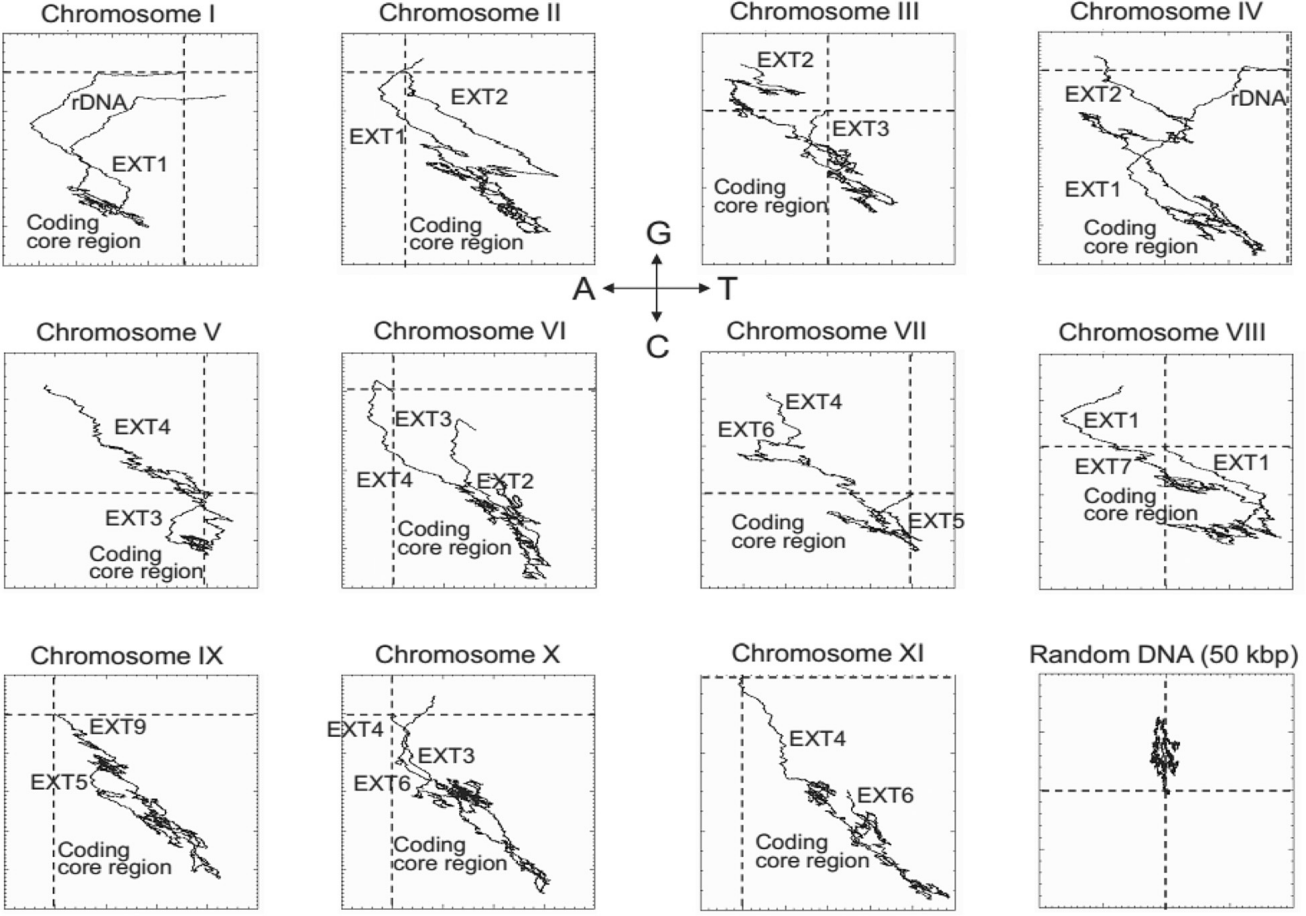
Genomic landscape of *Encephalitozoon cuniculi*. GC and AT base composition skews were evaluated simultaneously using a 2D representation where the curve is drawn while reading the sequence from the 5-prime to 3-prime end. The course of the curve is obtained by going up for a G and down for a C and left or right whether an A or a T was read respectively. The evolution of GC and AT skews is similar for the 11 chromosomes of *E. cuniculi*. In a 2D representation, the core of the molecule presents a globular structure whereas the EXT and SUB sequences are characterized by G enrichment (in a core to telomere orientation). The AT skew is in an opposite way between EXT repeats and SUB region. The distal end of the SUB region is composed of repeats which present a T accumulation only. The compactness of the coding core regions of *E. cuniculi* chromosomes is apparently higher than thus observed in a random sequence (bottom right curve). Horizontal and vertical discontinuous lines indicate the zero position on the ordinate and abscise respectively. The two lines get cross at the first base in 5-prime of the consensus sequence deposited in the database

**Fig. 8:**
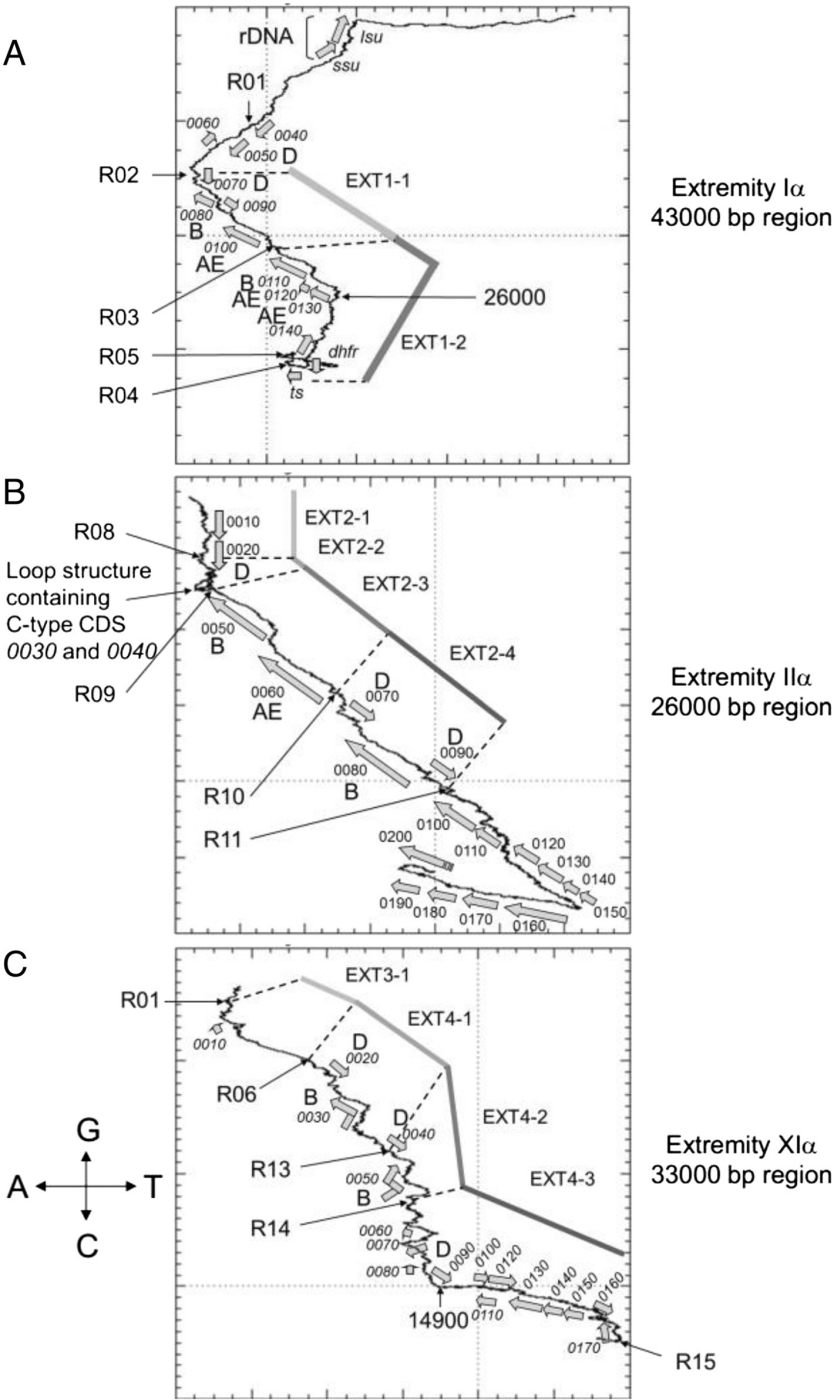
GC and AT base composition skews in EXT repeats and SUB regions are associated to transcription and replication. **a** *E. cuniculi* chromosome Iα extremity containing EXT1 repeat. Terminal regions (EXT and rDNA) present accumulation of G. The inversion of the AT skew takes place at R02 recombination site. This observation suggests that the region between R01 and R02 sites is related to the SUB region despite the presence of an *interD* gene close to R01. The accumulation of A is strongly associated with the presence of *interAE* and *interB* genes. The curve is in fact doing the revers in the gene poor region upstream of ECU01_0130 *interAE* gene. The G enrichment starts after the *dhfr-ts* gene cluster in EXT1-2 repeat. These knee points on the curve do not match with recombination sites. **b** *E. cuniculi* chromosome IIα extremity containing EXT2 repeat. R01 and R02 recombination site are not present in the consensus sequence of chromosome II actually present. The accumulation of A in EXT2 repeat is strongly associated with the presence of *interAE* and *interB* genes. It is also associated to *interD* genes in EXT2-4 but not in EXT2-1 sub-sequence. The *interC* gene in EXT2-2 displays a frame shift. It is therefore encoded by two putative CDS which may be two parts of a pseudogene. As all *interC* members, these CDSs are not associated to any base composition biases. The coding core region outside EXT2 also exhibits an accumulation of A up to CDS ECU02_0150. The inflexion of the curve between ECU02_0150 and ECU02_0160 CDS is marked by the inversion of both GC and AT skew. The same phenomenon is observed between ECU02_0190 and ECU02_0200 CDS. In both case genes are all transcribed in the same orientation. This suggests that origins of replication are present at these points in the IIα extremity. **c** *E. cuniculi* chromosome XIα extremity containing EXT4 repeat. An A + G accumulation is detected all over the EXT4 repeat starting at R015 recombination site. However, the curve shows several inflexion points due to local inversion of AT skew. The two *interB* genes present in this repeat are associated with these knee points. CDS names have been reduced to the last four digit numbers for a matter of space

The second approach consisted of the characterization of AT and GC skews along DNA strands in a 2D representation. This method is known as “DNA walk” [39]. It is a graphical method where the trails obtained for *E. cuniculi* chromosomes support the existence of a strong difference in the base composition between the core and the chromosome ends. Each chromosome core appears as a highly compact area with a wealth of loops (Fig. 7). The compactness of the curve is higher than in a random sequence measuring Brownian motion (Fig. 7). By contrast, the EXT and SUB regions display near-monotonic functions (Fig. 7). The progression in the sequence from the core to the extremity shows a G + A enrichment for most of the EXT blocks and a G + T enrichment for SUB blocks including the rDNA region (Figs. 7 and 8). The EXT-to-core frontier does not agree with the transition from a compact to a near-monotonic function (Fig. 8). This transition occurs between *dhfr* and *ts* genes within EXT1, in the case of Iα, Iβ and VIIIα extremities. In the case of IIα chromosome end, transition takes place within the coding core at ~ 20 kbp from EXT region. Local analysis of EXT repeats revealed that the DNA walk properties are tightly associated with *interAE*, *interB* and *interD* CDS (Fig. 8).

## Discussion

### First inventory of telomere-associated gene families in a microsporidian species

The *interAE*, *interB*, *interC* and *interD* gene families of *E. cuniculi* GB-M1 (strain I) currently offer the most complete view of subtelomeric gene repertoires in a microsporidian species. The *interAE* and *interB* full genes such as ECU04_1670 encode a protein precursor with an ER-signal peptide and a mature polypeptide containing a single N-terminal transmembrane domain and two conserved intracellular domains (Additional file 1: Figure S9). The *interC* genes encode polypeptides with 5 to 8 predicted C-terminal transmembrane domains associated with the DUF1686 domain (Additional file 1: Figure S9). No recognizable signal peptides were found in these proteins suggesting that the first transmembrane domain could be involved in endoplasmic reticulum targeting. Position of positively charged amino acids and TMPred predictions suggest that the N-terminal end of the InterC proteins is located in the cytosol. Most InterD proteins have 7 transmembrane domains. The physiological function of these genes remains unknown.

As shown in a study specifically devoted to *interB* gene family, some gene copies can be actively transcribed [33]. Genetic rearrangement among this gene family is less frequent than in the *interAE* or *interD* families. Indeed, *interB* multigene family contains only two truncated copies (ECU07_0050 and ECU09_2020 encoding the Y705_ENCCU peptide), one pseudogene in EXT9 and only one recombination site (R07). We also demonstrated that *interB* genes are conserved in four microsporidian species known to infect humans (*E. hellem, E. intestinalis, Vittaforma corneae* and *Anncaliia* (*Brachiola*) *algerae*), but not in species that parasitize insects or fish [33]. The finding of such genes in *A. algerae* is notable because (i) this species is a parasite of mosquitoes and accidentally infect humans [40, 41], and (ii) its genome is more complex (15–20 Mbp) than the one of *Encephalitozoon* species [33]. Hence, the *interB* repository would be of relatively ancient origin and might play a key role in the adaptation to the invasion of mammalian hosts.

The present work revealed the predominance of genes assigned to the *interD* family. The *interD* coding sequences are all oriented in the opposite direction of *interB* genes in EXT repeats. Many recombination sites have been mapped within *interD* genes and several CDS represent pseudogenes. ORF fusion is also observed in the *interD* multigene family. Interestingly, the promoter region of the consensus copy (D1 part of the gene) is predicted to contain a CCAAT box upstream of the TATA box, a rare case among all other *E. cuniculi* genes. Thus, a transcriptional control involving specific interactions of the CCAAT box with a heteromeric complex similar to the yeast HAP2/HAP3 complex [42] may be considered for *interD* gene regulation. The two candidate CDSs for this complex are ECU03_1510 (for subunit 2 or B) and ECU10_0260 (for subunit 3 or C). ECU03_1510 encodes a truncated HAP2 homologue (123 aa *versus* 265 aa in *S. cerevisiae*).

The *interC* and *interD* gene repository can be found in other *Encephalitozoon* species sequenced so far according to MicrosporidiaDB. Noteworthy some members of the *interC* gene family are present in the chromosome coding core region (Additional file 1: Figure S10). This is also the case of some multigene families coding for variable surface antigens in protozoan parasites, e.g. var family in *Plasmodium falciparum* [43], *vesa* genes in *Babesia bovis* [31] or *vsg* family in *Trypanosoma brucei* [44]. The *interC* family is only represented by 7 genes at chromosome extremities. We notice that four of them are presenting a frame shift mutation. As many *interC* genes are present in the coding core of chromosome IX (8/19), X (2/19) and XI (2/19), it is conceivable that the existence of chromosome core-associated loci reflects an early phase of the expansion of a subtelomeric gene family. This view is underpinned by the specific organization of *interC* genes at internal loci which are present by pair in inverted orientation. The transfer of genes towards proximal subtelomeric domains might have taken place afterwards as suggested by the presence of the ECU07_01800 gene at EXT-to-core transition of VIIβ extremity. Then, the duplication of EXT regions responsible for the amplification of *interAE*, *interB* and *interD* multigene family would also have enhanced *interC* gene copy number at chromosome extremities. However, we cannot fully exclude the alternate hypothesis involving a subtelomere-to-core gene transfer, as a prerequisite to the preservation of an essential function.

The sequencing of several *Encephalitozoon* genomes revealed the high level of conservation of gene content and structure of chromosome coding core region [34–36]. MicrosporidiaDB database confirmed the presence of the four gene families in *E. intestinalis*, *E. hellem* and *E. romaleae* genome. Other telomere-associated gene families were also described in these species [35]. The EXT region remains much less extended in the genome of *E. intestinalis* [34]. Protein features (Additional file 1: Figure S9) strongly suggest the products of these gene families could interact between each other. For a better understanding of the adaptation of microsporidia to their host organisms, it will be especially important to determine whether expression of some of these genes is required for immune evasion and/or host cell invasion.

### Chromosome regionalisation in *E. cuniculi* genome

The chromosomes of *E. cuniculi* GB-M1 show three regions that share little to no homologies between each other: the coding core, the EXT repeats and the SUB region. EXT repeats and SUB region persist on all chromosome ends of the small genome of *E. cuniculi*, which contrasts with the low redundancy of chromosome core-associated genes and the lack of large repetitive elements related to transposons. The whole length of DNA allocated to chromosome ends reaches about 450 kbp, representing ~ 15 % of the haploid genome size. The SUB region is 20 kbp large encompassing one rDNA unit and ending with the telomere. The size of the chromosome regions comprising the EXT repeats varies from 3.5 to 23.8 kbp. The gene density is high in EXT repeats. Genetic information appears highly compartmentalized in *E. cuniculi* genome.

Base composition at chromosome ends is significantly different from the coding core region. Chromosome extremities are characterized by a high GC content (Fig. 5). This GC enrichment is associated with a GC skew that extends from the EXT-to-core transition to the end of the rDNA region (Fig. 8a). Base composition of the EXT and SUB sequences does not depend on gene content. It might result from strand asymmetries in the frequency of mutations which could be related to either replication and/or transcription. Replication and transcription are intrinsically asymmetric processes that could lead to biased mutation rates between leading and lagging strands or template and non-template strand respectively. In prokaryotes, where transcription and replication often occur in the same direction, the leading replicating strand presents an excess of G over C and of T over A [45–47]. In eukaryotes, the existence of similar replication-associated asymmetries has been established both in the human genome and in the yeast centromeric and telomeric regions [48–51]. In fact, the lagging strand is temporarily single-stranded during the replication process and is more likely to undergo deamination of methylated cytosine which will be mutated into thymine [52]. The same strand asymmetry has been observed in the polycistronic region of the genomes of kinetoplastidae [53] but transcription can be associated with a different transition rate asymmetry in genomes where transcriptional units are organized in a more standard way [50]. Processes such as default in the repair machinery are then invoked to explain differences between replication- and transcription-associated base transition rates [50, 52]. Other DNA associated mechanisms do influence base composition as well. Meiotic recombination which is a symmetrical process influences GC content at hot spots of recombination in eukaryotic genomes [54–57]. The process driving W (AT bases) - > S (GC bases) transition in regions surrounding meiotic recombination sites is called GC-biased gene conversion (gBGC).

The strand-asymmetry found in the SUB sequence, between the EXT repeats and rDNA region, is characterized by an excess of G and T on the strand read in that orientation (Fig. 8). According to transition rate models described above, this asymmetrical skew pattern is indicative of constitutively active replication origin for many generations. It suggests that no origin of replication is present within the SUB region or in the telomeric region. The last origin of replication should be located in either an EXT repeat or the coding core part of the chromosome. We observed an inversion of the AT skew between EXT and SUB regions. The excess of A could result from the activity of the RNA polymerase. The G + A over C + T enrichment is strongly associated with CDS except for the *interC* genes that behave as the coding-core regions (Fig. 8b). Chromosome IIα extremity strengthens this view because the AT and GC skews extend far away beginning of the EXT2-4 repeat in the coding region where all genes are transcribed in the same orientation and skews are inverted as genes are transcribed in the opposite way (Fig. 8b). The origin of replication responsible for the duplication of the IIα extremity during S phase might then be located between CDS ECU02_00150 and ECU02_160.

Strand-asymmetry in *E. cuniculi* chromosome ends is in agreement with the model that replication and/or transcription are acting all over the region in a centripetal way. Replication may then continue up to the telomere past the rDNA unit. The absence of GC skew in the part of the molecule might be related to its origin. Although available only for chromosome I ends, the terminal sequence extending downstream of rDNA is characterized by heterogeneous degenerated tracts of telomeric DNA [11]. Strand-asymmetry DNA walk profiles strengthen the idea of a regionalisation of the end of *E. cuniculi* chromosomes and helps to differentiate the subtelomere (i.e. the SUB region) from a subterminal region corresponding to the EXT repeats.

### Chromosome regionalisation reflects cellular organization

Telomere bouquets are common structure in eukaryotes. In yeast and *Plasmodium*, these structures where the telomere is attached to the nuclear membrane exist all along the cell cycle [58–60]. Nuclear periphery has emerged as an essential aspect of gene regulation during interphase [61, 62]. This compartment is enriched for silencing factors such as Sir2, a NAD-dependent lysine deacetylase involved in the formation of heterochromatin [63–65]. In the absence of insulator, the effect of telomere-associated protein complexes (telosome) can extend over several kbp [66, 67]. Repression of genes in sub-terminal regions is called the Telomere Position Effect: TPE [63, 68, 69]. Many components of the telosome are present in *E. cuniculi* (e.g. Sir2: ECU03_0460). This mechanism may influence the expression of EXT genes which are very likely polII genes. Indeed, Dia et al. have shown that the *interB* genes are not transcribed with the same efficiency in *E. cuniculi* [33]. Furthermore, the SUB region in *E. cuniculi* could be considered as heterochromatin for several reasons: 1) it contains the rDNA region, a usual component of constitutive heterochromatin (Fig. 1); 2) it codes for very few genes and 3) significant methylation of the cytosine in the heterochromatin region would increase the possibility of C- > T transitions.

The telomeres are associated with DNA repair and recombination factors which are recruited by members of the telosome [70–73]. These factors are essential to Double Strand Breaks (DSB) repair processes at chromosome extremities. These DSB are deleterious as they are associated with the loss of the telomere and correct anchoring of the chromosome extremity to nuclear periphery. Repair mechanisms of double-strand break (DSBR) involve homologous recombination (HR) or non-homologous end joining (NHEJ) [74, 75]. *E. cuniculi* lacks nearly all NHEJ pathway associated proteins [76]. The Break Induced replication pathway (BIR) is a DBSR that depends on homologous recombination at micro-homology domains and enables telomere capture [77–79]. In yeast, telomere capture would lead to recombinant chromosomes that share similar structure and favour the formation of amphimeric molecules [12]. Mosaic organisation and gradient diversity of EXT regions could be explained by DSBR such as BIR. Cluster of telomere at nuclear periphery would help this phenomenon. Interestingly, this mechanism could also be at the origin of many CLP observed in *E. cuniculi* isolates [9, 10].

**Fig. 9:**
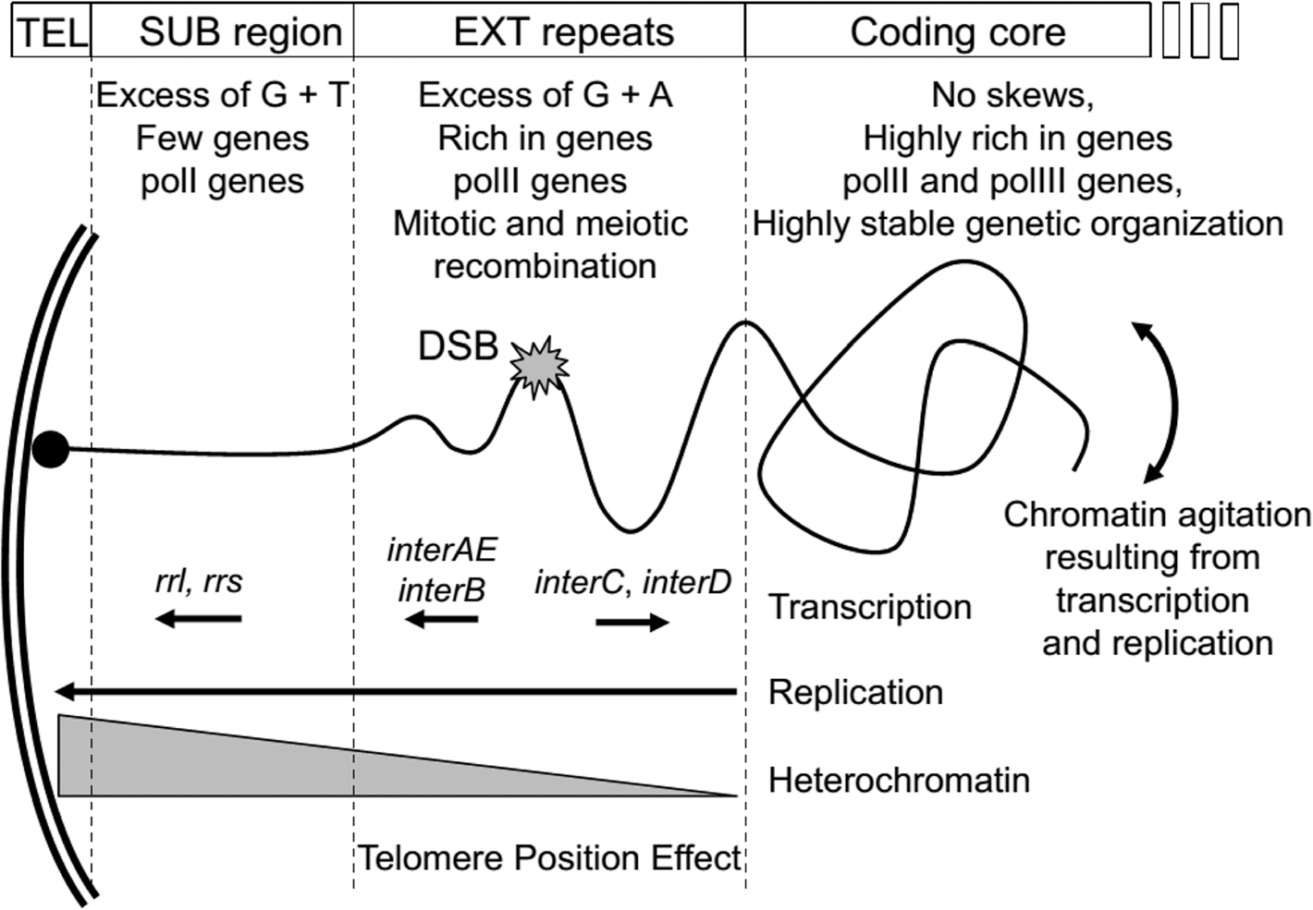
Regionalisation of the DNA molecules depends on several biological processes. Four regions could be found in *E. cuniculi* genome (top of the schema). We summarize all the possible mechanism that may influence genetic structure and base composition of *E. cuniculi* chromosome extremities (Transcription, replication, level of heterochromatin). The SUB region corresponds to the subtelomere and is related to heterochromatin state. The EXT repeats are presenting a mosaic organisation in *E. cuniculi* genome. They contain genes that might experience Telomere Position Effect (TPE). We suggest that continuous tensile and bending forces applied on the EXT by the movement of the coding region are responsible for Double Strand Breaks (DSB). The predicted “ductility” of the DNA need to be further tested. This model can be applied to other eukaryotic genomes with specific properties of each organism. Gene content and protein complexes such as telosome, DNA or RNA replication might deeply influence the presence of sub-terminal region in other genomes

The regionalisation of *E. cuniculi* chromosomes is the result of multiple interactions at chromosome extremity (Fig. 9). The telomere recruits the telosome, a protein complex that is rich in proteins involved in DSBR. Mosaic organisation of chromosome ends suggests that EXT repeats are more susceptible to DSB. We consider the resistance to tensile and bending of the DNA molecule to model and assess the presence of the sub-terminal region in *E. cuniculi* (Fig. 9). On one side of the EXT regions, transcription and replication acting continuously in the coding core of the chromosome are generating a continuous agitation of the DNA molecule. On the other side, the subtelomeric region (SUB) is attached through the telomere to the nuclear periphery and protected from tensile and bending by the compactness of constitutive heterochromatin organisation. Ductile overload fracture will occur as forces are applied to EXT repeats causing permanent distortion and subsequent fracture (Fig. 9). This model might be universal suggesting that telomeric, subtelomeric and sub-terminal regions could in fact be distinguished among eukaryotic chromosomes. Further analysis will be essential to evaluate their relative presence in other genomes. Genetic importance of chromosome extremities emphasizes the fact that great effort will be necessary in the future to characterize more carefully these regions during whole genome sequencing efforts.

## Conclusions

Analysis of the *E. cuniculi* chromosome extremities revealed novel properties of sutelomeric regions: a mosaic organisation of the sequence, a GC content bias, presence of putative coding sequences (CDSs) that were organized in multigene families that seemed specific to human microsporidia and recombination sites as borders of blocks of repeats. The organization of the chromosome ends in *E. cuniculi* has been compared with those of reference genomes such as that of yeast, human or malaria parasites. In many cases, sequencing of chromosome ends revealed a mosaic of specific interchromosomal segmental duplications as observed in *E. cuniculi*. The potential importance of the multigene families described is also discuss as in many human parasites where repeated sequences in these regions encode factors involved in host-cell interaction and/or immune escape mechanisms.

The findings reported here on this important model genome for a human parasite may change our understanding of chromosome ends in eukaryotes, their biology, evolution and relationship to pathogenesis.

## Methods

### Purification of *Encephalitozoon* spores and DNA extraction

*Encephalitozoon cuniculi* GB-M1 isolate was grown *in vitro* in either Madin-Darby canine kidney (MDCK) cells or human foreskin fibroblasts (HFF) at 37 °C, as previously described [80]. Culture supernatants containing parasite spores were pooled then subjected to centrifugation at 3,000 × g for 10 min. The resulting pellet was further treated 15 min at 50 °C with 1 % SDS to eliminate residual host cell material. Spores were then washed and stored in phosphate-buffered saline (PBS) at 4 °C until use.

DNA extraction was performed using the Elu-Quick kit (Whatman Schleicher & Schuell (GE HealthCare). About 10^9^ spores were suspended in 120 μl of NaI-containing lysis buffer and incubated for 15 min at 50 °C. After mixing with 240 μl of NaClO_4_ binding buffer, 60 μl of glass milk solution was added and the suspension was gently shaken at room temperature for 10 min then centrifuged at 7,000 × g for 30 s. The glass milk was washed in 1 ml of washing buffer following manufacturer recommendations. DNA was suspended in 20 μl of distilled water. Aliquots of 1000 X diluted solution were used for PCR amplification.

### PCR amplification and DNA sequencing

PCR primers that were positive for PCR and long-PCR amplifications are described in Additional file 2: Table S1. Primers designed in the chromosome coding core were named according to the consensus sequence ID and extremity (a or b). Primers at chromosome ends were called according to the sequence block ID identified by miropeat. Long-PCRs were performed using the Triplemaster PCR system (Eppendorf). Amplification started with 10 cycles of a two-step program: 20 s at 94 °C plus annealing/elongation at 68 °C with time depending on the size of the amplicon (3–12 min); followed by 20 cycles during which each annealing/elongation time was increased by 20 s.

Sequencing of 6 large amplified DNA fragments was performed using DNA library construction after partial digestion with *Sau*3AI enzyme (New England Biolabs - NEB) for 30 min at 37 °C then 20 min at 65 °C, except for LK09a3/Lr02r2 amplicon that was digested with *Sty*I (NEB) because of paucity of *Sau*3AI restriction sites. *Sau*3AI fragments were cloned into a *Bam*HI dephosphorylated pUC118 vector, whereas *Sty*I fragments were cloned into a CIP dephosphorylated empty pGEM-T (Promega) linearized by *Sty*I. Sizing was performed between 0.5 and 1.5 kbp. The number of sequenced subclones per amplified fragment was 25 for Lr05debrev/Lr02r1-2 (4.5 kbp), 34 for Lr05debrev/Lr13dir3 (2.3 kbp), 47 for Lr08rev/Lr02r1-2 (4 kbp), 16 for LK09a/Lr02r1-2 (10 kbp), 63 for LK09a3/Lr02r1-2 (4 kbp), and 23 for LK03b5/Lr02r1-2 (11 kbp). Pair ends DNA sequencing (Sanger method) was performed by Cogenics (Beckman Coulter). Sequence assembly was performed using DNABaser software (Heracle BioSoft SRL). Lr05debrev/Lr02r1-2 and Lr05debrev/Lr13dir3 sequencing provide missing information at SUB-EXT2 transition (Acc N° HG967527). LK09a/Lr02r1-2 and LK09a3/Lr02r1-2 sequences give IXα organization (Acc N° HG967528). Sequencing and assembly of LK03b5/Lr02r1-2 subclones provide two consensus sequences, a short version (Acc N° HG380753), and a longer one including EXT4-1 region (Acc N° HG967526). All final consensuses were used as bait, using reads from the genome project as prey to confirm new sequence organizations. Sequencing of Lr08rev/Lr02r1-2 was used to study diversity in the EXT4-rDNA transition.

### Bioinformatics analysis

Miropeat software [81] was compiled on UNIX Solaris and LINUX operating system. The source code is available at http://genome.wustl.edu/pub/software/miropeats. Known *E. cuniculi* chromosome consensus sequences were compared to each other through the use of three-sequence combinations (Additional file 1: Figure S1). Blastall program [82] was downloaded from NCBI ftp server or used through the web portal (www.ncbi.nlm.nih.gov/blast). GeneFizz server was used for analysing base skews along DNA strands (web site no longer available) [37]. The DNA walk algorithm was an automaton reading the sequence from 5’ to 3’ [39] and was implemented with a movement going from the (0.0) coordinate toward up, down, left and right for G, C, A and T bases, respectively. Graphic representation was accomplished using GNUPlot software or IDL data analysis and visualization software (Exelis).

### Ethical consideration

The manuscript does not need any ethical approval.

## Description of Additional files

### Miropeat analysis

The *Encephalitozoon cuniculi* genome analysis using Miropeat software detected large gap-free repeats at the end of the chromosomes (Additional file 1: Figure. S1). Overlapping distal repeats were separated in fifteen types of repetition units (r01 to r15, Additional file 1: Figure S2). Five segments of unique type (be1 to be5) extend between some repeats on three chromosomes, the largest one (be3, 9 kbp) being intercalated between r02 and r09 on chromosome VIII. The two ends of every individual chromosome (Σ) were designated as Σα and Σβ, referring to the 5’ and 3’ ends of the Watson strand of the sequence deposited in the database, respectively.

The EXT and “r” repeats are generally present once per extremity (Additional file 1: Figure S1). Three chromosomes have large symmetrical organisation of their extremities: EXT1, r01-r02-r03-r04-r15 on chromosome I (37 kbp of internal repetition), EXT3-EXT4, r10-r07-r08 on chromosome VI (7 kbp of internal repetition) and EXT1, r02 on chromosome VIII (about 4 kbp of internal repetition). The r02 repeat is the most frequent sequence found after Miropeat analysis (11 copies). It is not entirely repeated as it is present. The largest form is located at extremities Iα, Iβ, IVα and VIIIβ (about 10 kbp). Some sequence polymorphisms between the copies of the different chromosomes can be observed at 3 kbp from the distal extremity (5 prime of the sequence). It can be correlated to the beginning of EXT1. Shortest r02 copies at extremity Vα, VIIα and Xβ are corresponding to this first 3 kbp region. The VIIIα copy is identical to the end of chromosome I version. It differs from VIIIβ with one indel at 700 bp from the 3 prime end. The IIα r02 copy is identical to VIIIβ but the first two kilobase pairs are missing. Short internal sequences of r02 are also found at extremity IIIα and IXβ. The repeats r01 (IVα), r05 (VIβ and VIIα), r07 (VIIβ, Xα), r08 (Vβ) and r10 (VIα, VIβ, XIα) are also presenting deletion in their 5 prime extremities compared to the largest type repeat. The copy number of “r” repeats varies from 2 to 6: r09, r11, r12, r13, r15 -> two copies matching perfectly expect one difference in r11; r01, r03, r04, r06, r14 -> found three time in the genome (r01 and r14 are presenting polymorphisms); r05 and r07 -> five copies (r05 is the most polymorphic “r” repeat); r08 and r10 - 6 copies (r08 is very conserved). The r05 repeat is the only one which is present twice at one extremity (VIβ).

Partial r02 segments at IIIα, Vα, VIIα, VIIIα, IXβ and Xβ are likely due to artificial truncations. However, it should be noted that a shortened repeat version cannot be considered as an artefact when found in internal position. This is the case of two small r05-type segments bordering an r14 copy on VIβ, the other r05 copy flanked by a unique DNA sequence on extremity VIIα and the three r14 copies (on IIα, VIα and VIIα). A round of comparison at the gene content and sequence levels using BLAST algorithm was necessary to assign definitively “r” and “be” sequences to EXT blocks. For example, EXT4 covers the series r07-r08-r12 (17.7 kbp) on XIα but is apparently restricted to r08-r12 (14.2 kbp) on Vβ or only r07-r08 (3.4 kbp) on Xα. Short unique “be” segments may be associated with one or two adjacent repeats, e.g. r05-be1-r14 for EXT2 and r13-be2-r05 for EXT5. Only the be3 segment on VIIIβ persisted as a single copy but, waiting for information about missing sequences, this segment was also retained for further analysis and designated as EXT7.

### Miropeat – EXT correspondancy

Every complete *E. cuniculi* subtelomere should present a conserved region encompassing an rDNA unit, but the r01 repeat may not be the only rDNA-associated sequence since the r01-r02 transition was only defined by a small gap on chromosome IV (compared to chromosome I). It was useful to ascertain whether the conserved subtelomeric region may overlap r02. Two breakpoints of homology with different chromosomes were detected within the largest r02 version (10 kbp on chromosome I). They are the mark for transition between the subtelomere and the EXT subterminal repeats. Size estimations from chromosome I give two subtelomeric block of 14 kbp for SUB-1 (10.5 kbp for r01 + 3.5 kbp for 5’part of r02) and 2-kbp for SUB-2.

This is well exemplified by EXT1. Each being clearly anchored with an SUB-2 block, the three EXT1 blocks on Iα, Iβ and IVα are the most representative of complete junctions of subtelomeres with chromosome cores. Every EXT1 block starts at the second r02 breakpoint (R02 recombination site) and extends on either two or three other repeats (r03-r04 on VIIIα, r03-r04-r15 on Iα and Iβ). The r02-r03 boundary corresponds to a loss of homology between Iα and IVα (R03 recombination site). A specific feature of the r03 repeat (7.55 kbp) is represented by two CDSs (ECU01_0100 and ECU01_0130 in the case of Iα) sharing strong homologies in their 5’ coding and 5’ flanking regions (over ~ 700 bp). EXT1-2 overlaps r03 and r04, the transition between these repeats being only marked by a G insertion on VIIIα, at the middle of a 2.9-kbp region devoid of large ORFs and detected CDSs. The r04 and r15 repeats harbour several house-keeping genes. An aminopeptidase-dihydrofolate reductase (*dhfr*)-thymidylate synthase (*ts*) gene cluster is indeed specific to r04. Three other adjacent genes, encoding serine hydroxymethyltransferase, an ABC transporter and a protein related to the NAP/SET family are characteristic for r15, a repeat restricted to chromosome I.

A 700-bp stretch of AT-rich repeats at both ends of chromosome VI and on extremity XIα was not found in our sequences. Of artefact origin, this stretch should be removed from a further release of the genome sequence. The new IXα consensus (entry ID HG967528) also suggests that the first 54 bp of chromosome IX consensus (entry ID AL590451) should be discarded. It should be stressed out that although the number of EXT sub-blocks is close to that of the Miropeat-detected repeats or embedded sequences; the positions of the transitions provided by the two approaches do not necessarily match (Additional file 1: Figure S2). As a result of EXT determination, the number of recombination sites mapped between sub-blocks is reduced to 15, compared to the number repeat-to-repeat transitions.

### EXT repeat annotation

EXT1 repeat is present on six chromosome ends (including both ends of chromosomes I and VIII, Additional File 1, Figure S5A). R01 and R02 recombination sites have been precisely mapped. They are both located in an interD gene. The SUB region downstream of R01 (SUB-1) harbours one transcription unit for the precursor of the large (*lsu*) and small subunit (*ssu*) rRNAs. The R03 recombination site is present six times in the genome. It is a hot spot of recombination. The conserved left-hand part of the recombination is associated to four different sequences located at the right side of site. The size of EXT1-1 is presenting a variation of more or less 1 kbp depending on the location of the transition with the four centromere-proximal sequences. The shortest version of EXT1-1 was found on chromosome VIII. The longest one was characterize on chromosome I and II.  The EXT1-1-to-EXT7 transition is inside one* interAE* CDS (ECU01-0100 on Iα and ECU08_2070 on VIIIβ). No homologies were found between the different “right” sequences.EXT2 repeat is present on four chromosome ends (Additional File 1, Figure S5B). At chromosome core transition, the position of the R10 breakpoint failed to be localized precisely because of multiple sequence combinations and presence of degenerated *interAE* gene copies. On the other side, the attachment to SUB-2 in IIa and IVb chromosome ends was validated by PCR experiments and sequencing of the Lr05debrev-Lr13dir2 PCR. Two newly identified *interB* genes (newECU02 and newECU04) were previously partially described in a transcriptomic analysis performed by Dia et al [33]. These genes are associated with the R07 recombination site. The VIb EXT4-EXT2 junction results from a fusion between R08 and R13 sites. The IIIb1 is presenting the same organisation except that the interC can now be fully transcribed. We have no information but we know the the newECU03 *interB* adjacent gene is expressed at a high level [33]. The position of the R10 breakpoint failed to be localized precisely because of multiple sequence combinations and presence of degenerated *interAE* gene copies.EXT3 repeat is present on three chromosome ends (Additional File 1, Figure S5C). EXT3-1 is found in 12 extremities but the distal part of EXT3-1 in associated to EXT3-2 only at chromosome IIIb1, IIIb2 and Xa extremities. All CDSs of Xα diverge significantly from those of IIIa (note a complete interC gene on Xα) but a same gene order is preserved. Extremity IIIa and IIIb2 are very similar, providing a nice evidence that chromosome III is an amphimeric molecule including EXT3-2, EXT3-1 and SUB region. The centromere-proximal region of EXT3-2 which is associated to the R09 recombination site is highly similar to EXT2-2. As recombination site are identify by their distal part, we identify R09a and R09b version of that hot spot of recombination. R01 and R06 recombination site are associated with an interD genes.  The IIIb2 extremity is carrying the second copy of the EXT10 repeat.EXT4 repeat is present on seven chromosome ends (including both ends of chromosomes VI and XI, Additional File 1, Figure S5D)). Linkage to SUB-1 occurs via EXT3-1. XIa and Vb can be paired and possess the longest form of EXT4 (EXT4-3 is partly shown in the schema). R10 was grossly mapped to the end of an *interD* gene prone to various recombination events. EXT4-2 is a short sub-sequence that shares strong homology with EXT5-1 and the beginning of EXT1-1. A unique EXT8 block extends upstream of EXT4-2 on VIa. The R08/R13 fusion was already described above for EXT1. The R13 site is also involved in the EXT4-EXT6 transition.EXT5 repeat is present on two chromosome ends (including both ends of chromosome IX, Additional File 1, Figure S5E). EXT5-1 shares 80% identity with EXT1-1, the divergence between these sub-blocks resulting from point mutations and Indels. The EXT5-1 sub-sequence is also associated to EXT2 full organization at IIa and IVb. We consider that these two chromosome ends may carry the original form of EXT5-1 as R07 recombination site at that extremities is associated to the full copy of* interB* genes NewECU02 and NewECU04. These genes are only weakly expressed in *E. cuniculi* [33]. The organisation of IXα was determined after sequencing of long-PCR products. The EXT9 sequence is unique in *E. cuniculi* genome but could be considered as a divergent copy of IXa extremity.EXT6 repeat is present on four chromosome ends (Additional File 1, Figure S5F). EXT6-1 is just upstream of EXT4-1 on VIIβ, Xα and XIβ. Despite that EXT6-2 contains no inter genes, its GC content is very high compared to chromosome core sequences (Additional File 1, Figure S7). The duplication of EXT6-2 characteristic for Xα and VIIIα is associated with an inversion (ARF and PRS6B indicate the two terminal genes encoding an ADP ribosylation factor and a proteasome regulatory subunit, respectively). This inversion may be a consequence of a more general genetic event, when considering that (1) on VIIβ, EXT6-1 displays an inverted *interC* gene and (2) on VIIIα, EXT6-2 is adjacent to EXT7-2 which appears to be of chromosome core origin (Additional File 1, Figure S5G). R18 was only approximately mapped as it is a hot spot of recombination. EXT7 repeat is present on one chromosome end (Additional File 1, Figure S5G). This unique sequence is upstream of EXT1-1 on VIIIα and is divided into two sub-blocks differing in both gene composition and GC content. EXT7-1 contains an *interD-interC* gene cluster similar to those in EXT2-2 and EXT3-2 while EXT7-2 harbours seven other CDSs including a gene for ribosomal protein L12 (RL12). GC enrichment is restricted to EXT7-1. The EXT7-EXT6 junction corresponds to the fusion of two recombination sites. Only R21/R19 sequence fusion could be precisely mapped.

## Additional files

Additional file 1: Figure S1. Miropeat analysis of *E. cuniculi* chromosomes. Miropeat analysis was performed on various set of sequences. The three-by-three analysis was performed to extract coordinates of “r” sequence blocks. **A**. Comparative analysis of the 11 *E. cuniculi* chromosomes. Most repeated sequences are associated to chromosome extremities. **B**. Three-by-three analysis confirming the existence of sequence block. Left: comparison of chromosome I, IV and VIII enables the identification of r01, r02, r03, r04 repeats. The r15 sequence was found by comparison of chromosome I with itself. Part of r02, r03, R04 and r15 repeats will composed the EXT1 sequence block. **Figure S2.** Miropeat – EXT correspondence. **A**. Superimposed distributions of repeated elements detected with Miropeat software (boxes r01 to r02), EXT blocks (coloured arrows 1 to 10). The r01 repeat includes one rDNA unit (red arrow) and r04 is ascribed to *dhfr-ts* (dihydofolate reductase - thymidylate synthase) gene cluster. Five DNA segments are of unique type (be1 to be5). Some EXT blocks may consist in the clustering of “r” and “be” sequences. EXT8 was created on the basis of BLAST homologies. **B**. *E. cuniculi* chromosome extremities have been arranged by referring to the conserved position of R01 recombination site. The R02 recombination is present at chromosome ends presenting an S-to-EXT1 or S-to-EXT5 transition. Hachured boxes correspond to missing regions in the Genoscope *E. cuniculi* genome release that have been reassembled in our study. EXT9 and EXT10 were completely characterized after specific cloning and sequencing of IIIβ and IXα ends, respectively. Highly conserved regions at all chromosome ends (telomeres and distal subtelomeric regions) are symbolized by a red dashed arrow on both sides of the schema. The “r” and EXT repeats are represented at their correct scale. The scale was not respected for SUB and coding core regions because of graphical reasons. **Figure S3.** Experimental validation of the mosaic structure described for *E. cuniculi* GB-M1 subtelomeres. Long-PCR amplifications were used to reconstruct chromosome ends. Three hypotheses for linking sequences were tested and are illustrated here: from EXT block to chromosome core (e.g. Lr12dir2/LKVβ), from rDNA to chromosome core (e.g. Lr02r1-2/LKIXα) and from rDNA to EXT block (e.g. Lr01-2/Lr07rev). **A**. general strategy based of PCR and long-PCR amplifications. **B**. Example of PCR amplification products. Agarose gel analysis of PCR products that are sometimes more than 15 kbp in size. The DNA size marker is Lambda DNA cut with *Eco*RI and *Hin*dIII. **C**. Relative positions of the primers, the R01 recombination site being taken as an origin. Primers sequences and orientations are given in Additional file 2: Table S1. **Figure S4.** Experimental validation of the two IIIβ extremities genetic organization in *E. cuniculi* GB_M1 strain. **A**. Schematic representation of IIIβ1 chromosome end organization deduced from PCR products and sequencing. The relative positions of the different primers were identified. Most primers were design from EXT repeats characterized in other chromosome extremities. Coordinates are given according to the current release of *E. cuniculi* chromosome III. **B**. PCR and long-PCR amplifications were used to reconstruct chromosome IIIβ extremities. Agarose gel analysis reveals PCR products that are sometimes more than 15 kbp in size. The DNA size marker is Lambda DNA cut with *Eco*RI and *Hin*dIII. **Figure S5.** Coding DNA sequences (CDSs) and recombination sites (R01 to R18) in EXT repeats distributed among the 22 chromosome ends (Σα, Σβ) of *Encephalitozoon cuniculi*. Each CDS is schematized by a large arrow showing the direction of transcription. New putative CDS deduced from *E. cuniculi* GB-M1 chromosome ends framework of reconstruction are represented by thick outlined shapes. To gain space, CDS names have been reduced to the last four digit numbers (e.g. 0040 on Iα and 0040 on IVα correspond to ECU01_0040 and ECU04_0040 in *E. cuniculi* genome databases, respectively). If available, Uniprot accession numbers for encoded hypothetical proteins are also given (Y103_ENCCU, Y110_ENCCU…). CDSs assigned to four multigene *inter* families (AE, B, C or D) are coloured in grey. Recombination hot spots, indicated by vertical dashed lines (“precise” sites) or hatched zones (“approximate” sites), determine the boundaries between EXT sub-blocks (e.g. EXT1-1, a sub-block of EXT1, is bounded by R02 and R03 sites). CDSs surrounded by dotted line are in the coding core sequence. CDSs with plain lines are EXT and SUB CDSs. Arrows without number are extrapolated from the present study. A. EXT1. B. EXT2. The thick horizontal bare indicate the region covered by corresponding Genbank accession number. C. EXT3. The thick horizontal bare indicate the region covered by corresponding EMBL/Genbank accession number. D. EXT4. E. EXT5. The thick horizontal bare indicate the region covered by corresponding Genbank accession number. F. EXT6. G. EXT7. **Figure S6.** Gradient complexity of EXT repeats from R01 recombination to EXT-to-core transition. Recombination have place in a tree-like structure respecting their relative distance to R01 recombination site. All EXT repeats and the 23 chromosome ends are represented in the schema. Recombination site fusion are represented by a discontinuous line. Duplication of R09 recombination site is indicated by a plain dark line. Interestingly, R08 and R13 sites were at the same distance from R01 when they get fused. **Figure S7.** Progressive or rapid GC % shift at EXT-to-core transition. EXT repeats are associated with high GC %. This observed shift is improved by the low GC % that is present at EXT-to-core transition (35 % GC in average). It was possible to measure the distance between the lowest GC % peaks at one chromosome extremity with the highest GC % optimum found in the adjacent EXT repeat (red line). We consider only the first EXT subsequence in that experiment. In fact, we observe that low GC % values were associated with some recombination sites. The slope of the GC % curve varies a lot between chromosomes. Four examples of the most extreme values are given in the panel. **Figure S8.** GeneFizz analysis of an *E. cuniculi* chromosome end (IIα) showing the relationship between open-closed transitions and recombination sites. **A**. An overview of a complete IIα organisation can be obtained by combination of different sequences (see bottom). CDSs are represented by boxes with arrows indicating their direction of transcription and are ordered from telomere (left) to chromosome core (right). Letters AE, B, C and D are applied to the members of the four multigenic families described in the present study. Name and Uniprot accession number are given for most CDSs. **B**. DNA of EXT repeats is getting melted at high temperature and offers an alternation of “open” and “closed” areas. Double stranded DNA (“closed” state) correspond to the null value. At the maximum, the DNA is fully melted, i.e. single stranded (“open” state). Corresponding colours are red for 72 °C, yellow for 73 °C, deep blue for 74 °C, light blue for 75 °C, violet curve without colour shading for 76 °C. The green line corresponds to the GC %. An increase of at least 3 °C is required for strand dissociation at some loci (*). Recombination sites, indicated by vertical dashed lines (“precise” sites) or hatched zones (“approximate” sites), determine the boundaries between EXT sub-sequences. Small vertical arrows at the bottom indicate the position that was taken into consideration to calculate the distance between open-to-closed transition and recombination site. **C**. Recombination sites R10 and R11 are associated with close regions. Corresponding colours, from 69 to 73 °C, are red, yellow, dark blue, light blue and violet. GC content curve is coloured in green. The R11 site is associated with a closed region in the proximal region. **Figure S9.** Description and putative structure of InterAE, InterB, InterC and InterD proteins. Protein description was recovered from Pfam database. We use one entry per multigene family product which was considered to be the most representative. We recovered both graphical representation of the protein and feature table. A schematic representation can be deduced from each Pfam description. We consider the distribution of positively charged amino acids (+) around the first transmembrane domain to assess the orientation of the InterC and InterD protein in the plasma membrane as they have no ER-targeting signal peptide. **Figure S10.** MicrosporidiaDB resources presenting genomic distribution of *interAE*, *interB*, *interC* and *interD* genes in *Encephalitozoon cuniculi* genome. Data were recovered of the GB-M1 strain that was used in the present study. They were also recovered for two other isolates EC1 and EC3. **A**. The *interAE* and *interB* genes were selected from the database based on their conserved Interpro domain IPR011667-UPF0329. Some truncated genes and pseudogenes were not detected. **B**. The *interC* genes were selected from the database based on their conserved Pfam domain DUF1686. **B**. The *interD* genes were selected from the database based on their conserved Interpro domain IPR019081-UPF0328. Some partial sequences were not detected. (PDF 1180 kb) Additional file 2: Table S1. Primer list used for the determination of chromosome ends structure in *Encephalitozoon cuniculi*. (DOC 85 kb)

## Abbreviations

rDNA: transcription unit of rRNA
rRNA: Ribosomal Ribonucleic acid
PCR: Polymerase Chain Reaction
BLAST: Basic Local Alignment Search Tool
CDS: Coding region sequence
PFGE: Pulsed Field Gel Electrophoresis
ORF: Open Reading Frame
NAD: Nicotinamid Adenine Dinucleotide
CLP: Chromosome Length Polymorphism
DSBR: Double Strand Break Repair
BIR: Break Induced Replication
