# Supplementary material for: Subtelomere organization in the genome of the microsporidian Encephalitozoon cuniculi: patterns of repeated sequences and physicochemical signatures
Source: BMC Genomics. 2016 Jan 7;17:34. doi: 10.1186/s12864-015-1920-7 (PMC4704409; doi:10.1186/s12864-015-1920-7)
Supplement: Additional file 1: Figure S1. — Miropeat analysis of E. cuniculi chromosomes. Miropeat analysis was performed on various set of sequences. The three-by-three analysis was performed to extract coordinates of “r” sequence blocks. A. Comparative analysis of the 11 E. cuniculi chromosomes. Most repeated sequences are associated to chromosome extremities. B. Three-by-three analysis confirming the existence of sequence block. Left: comparison of chromosome I, IV and VIII enables the identification of r01, r02, r03, r04 repeats. The r15 sequence was found by comparison of chromosome I with itself. Part of r02, r03, R04 and r15 repeats will composed the EXT1 sequence block. Figure S2. Miropeat – EXT correspondence. A. Superimposed distributions of repeated elements detected with Miropeat software (boxes r01 to r02), EXT blocks (coloured arrows 1 to 10). The r01 repeat includes one rDNA unit (red arrow) and r04 is ascribed to dhfr-ts (dihydofolate reductase - thymidylate synthase) gene cluster. Five DNA segments are of unique type (be1 to be5). Some EXT blocks may consist in the clustering of “r” and “be” sequences. EXT8 was created on the basis of BLAST homologies. B. E. cuniculi chromosome extremities have been arranged by referring to the conserved position of R01 recombination site. The R02 recombination is present at chromosome ends presenting an S-to-EXT1 or S-to-EXT5 transition. Hachured boxes correspond to missing regions in the Genoscope E. cuniculi genome release that have been reassembled in our study. EXT9 and EXT10 were completely characterized after specific cloning and sequencing of IIIβ and IXα ends, respectively. Highly conserved regions at all chromosome ends (telomeres and distal subtelomeric regions) are symbolized by a red dashed arrow on both sides of the schema. The “r” and EXT repeats are represented at their correct scale. The scale was not respected for SUB and coding core regions because of graphical reasons. Figure S3. Experimental validation of the mosaic structure described fo [file 12864_2015_1920_MOESM1_ESM.pdf]

A.

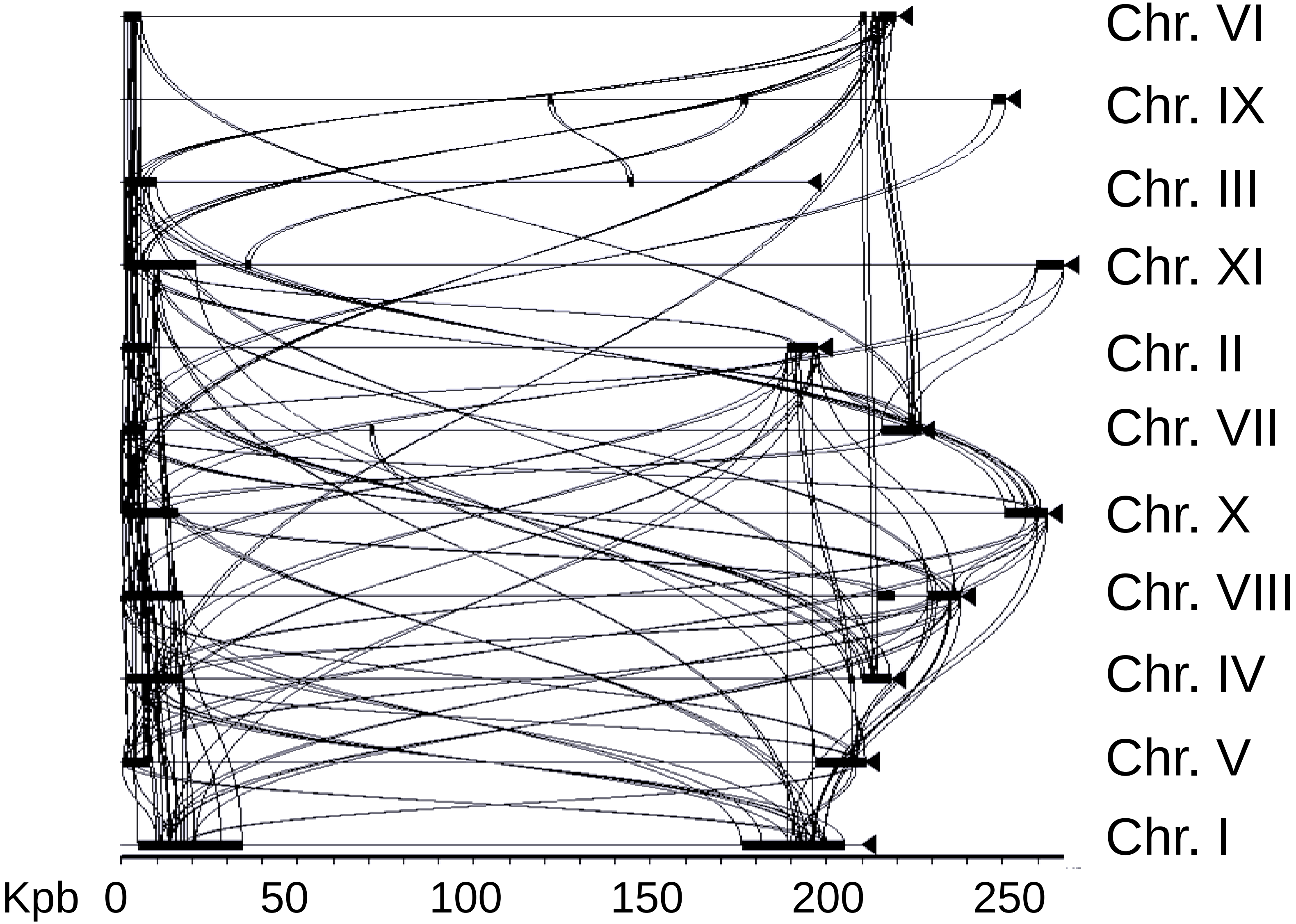

Supplementary Figure S1

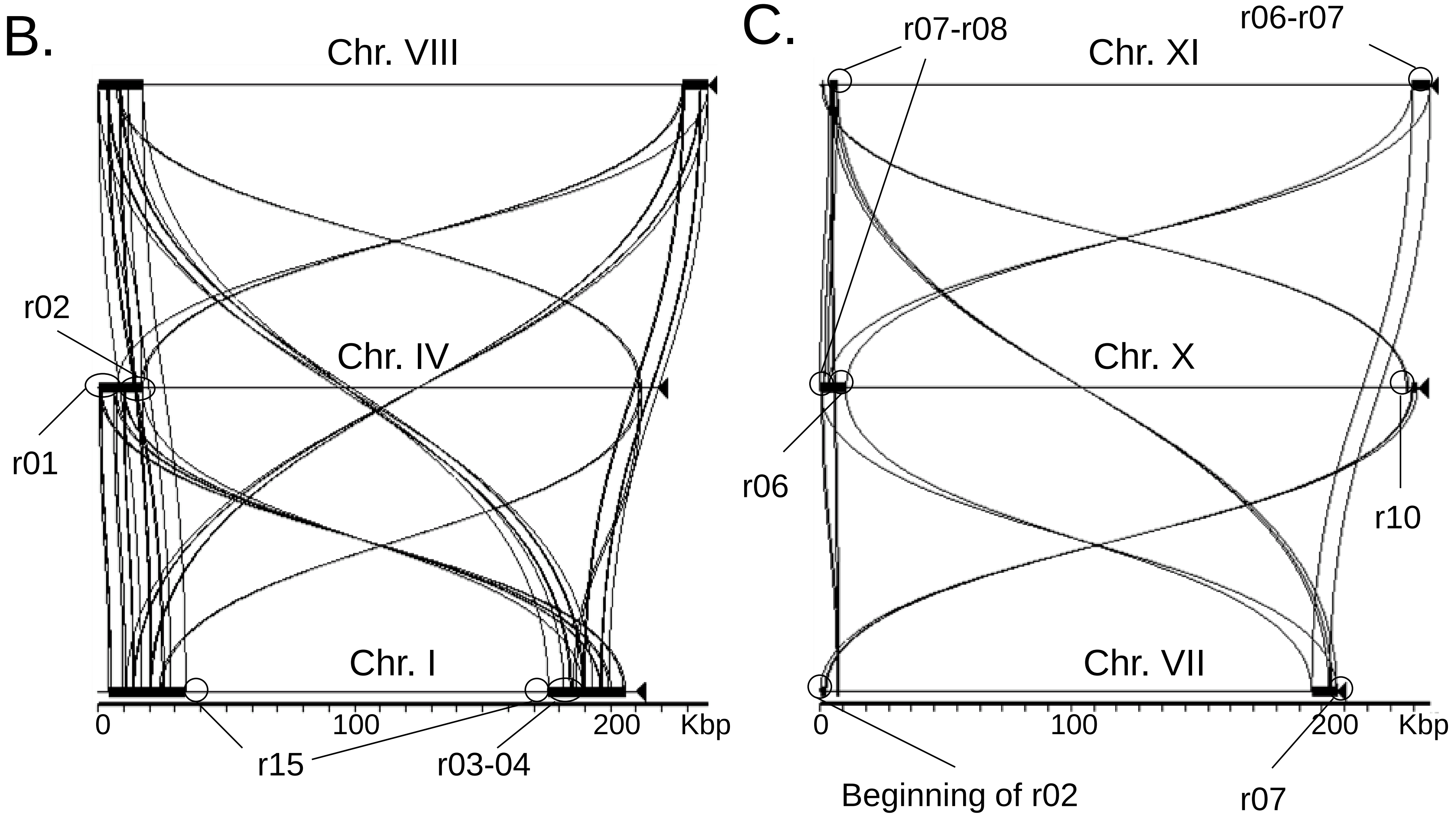

Supplementary Figure S1

Supplementary Figure S2

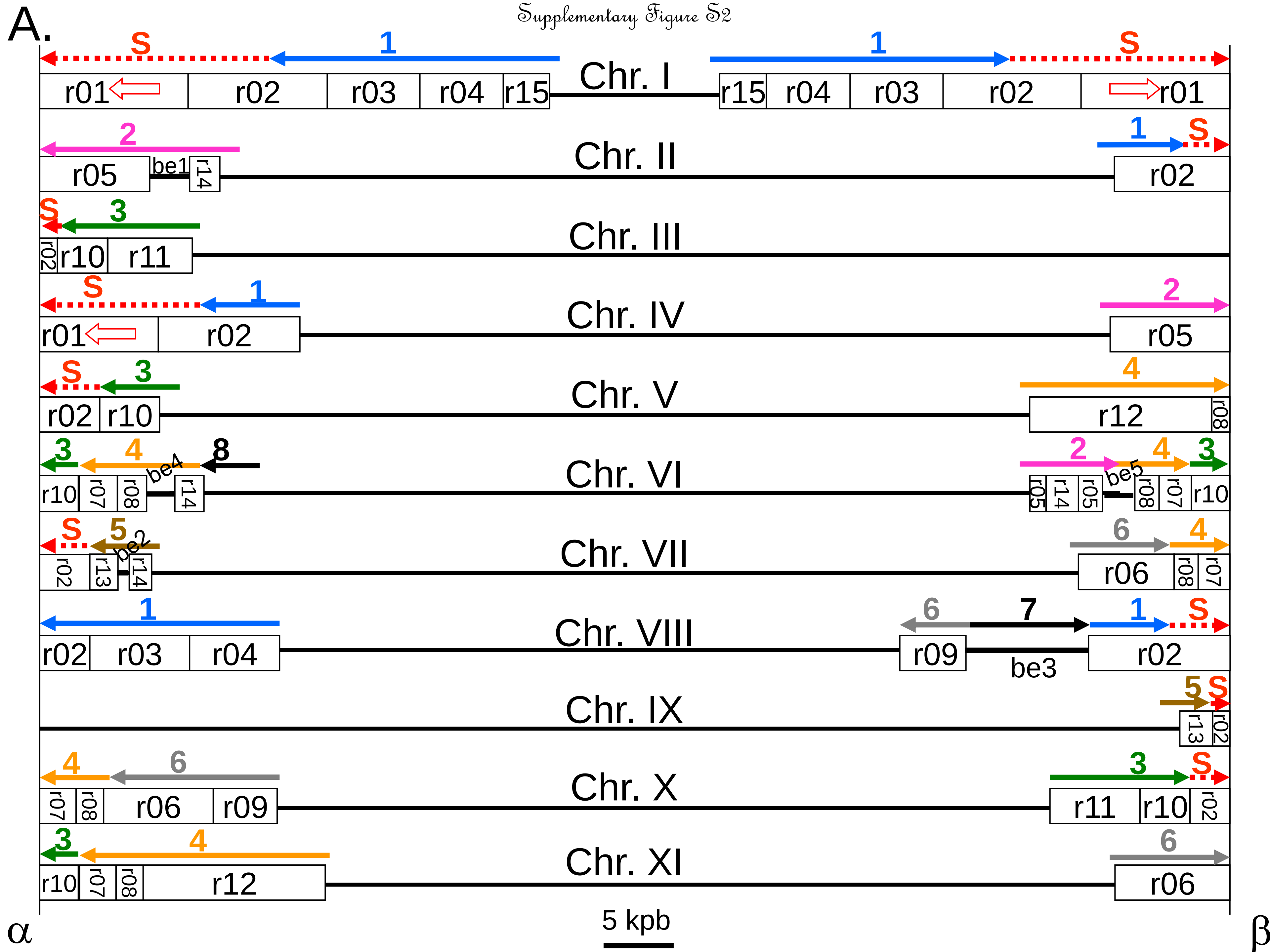

Supplementary Figure S2

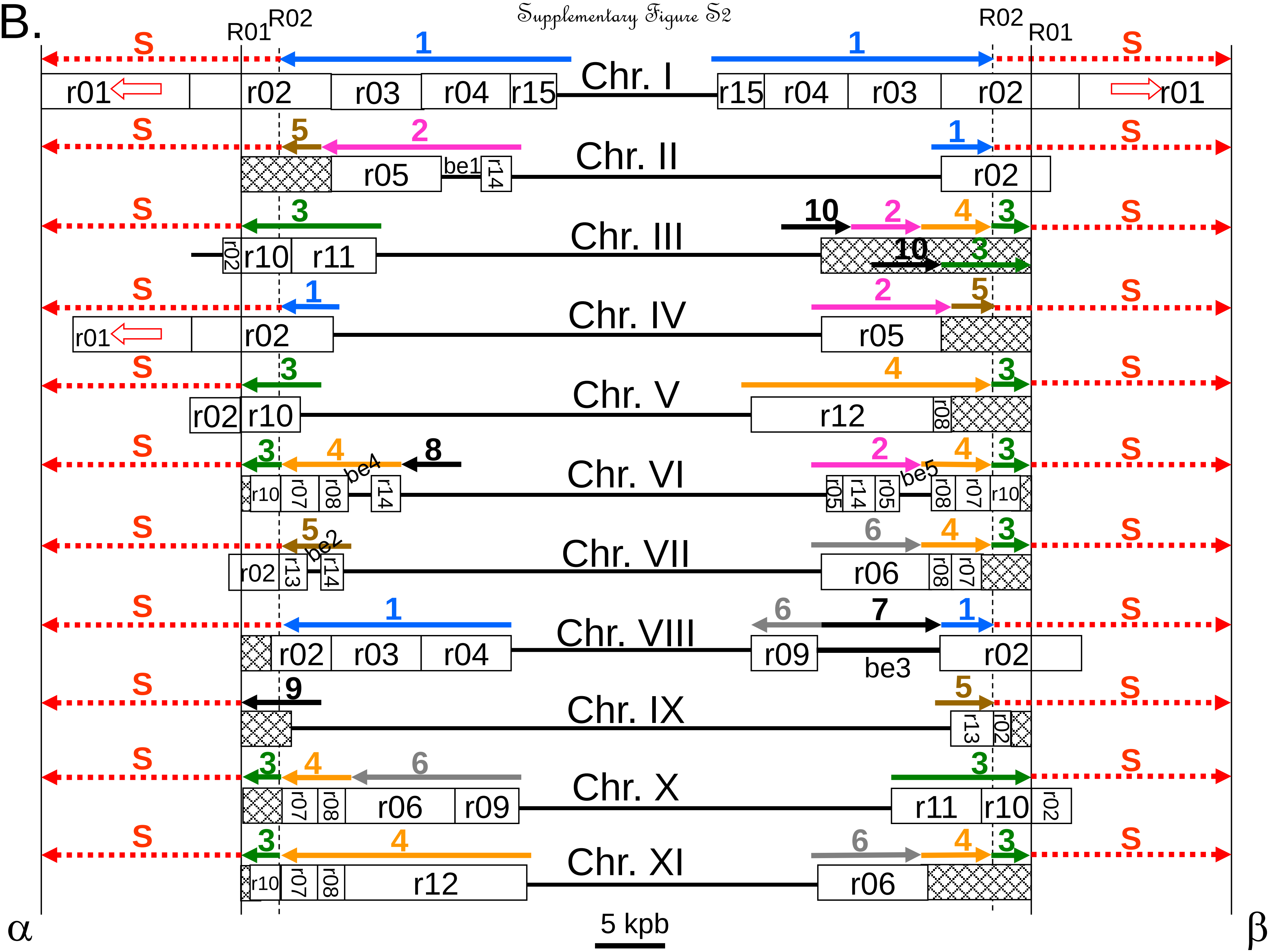

Supplementary Figure S3

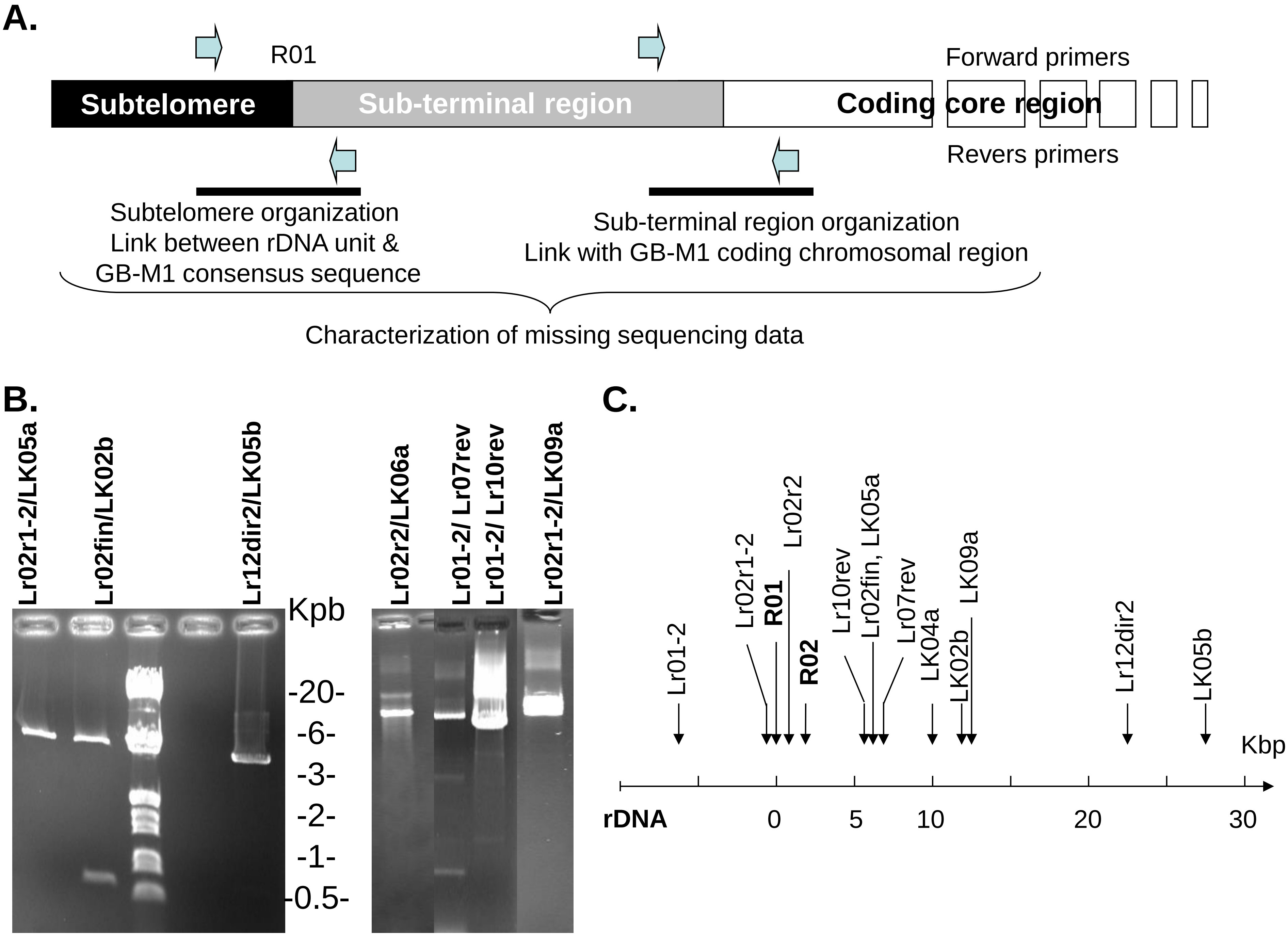

Supplementary Figure S4

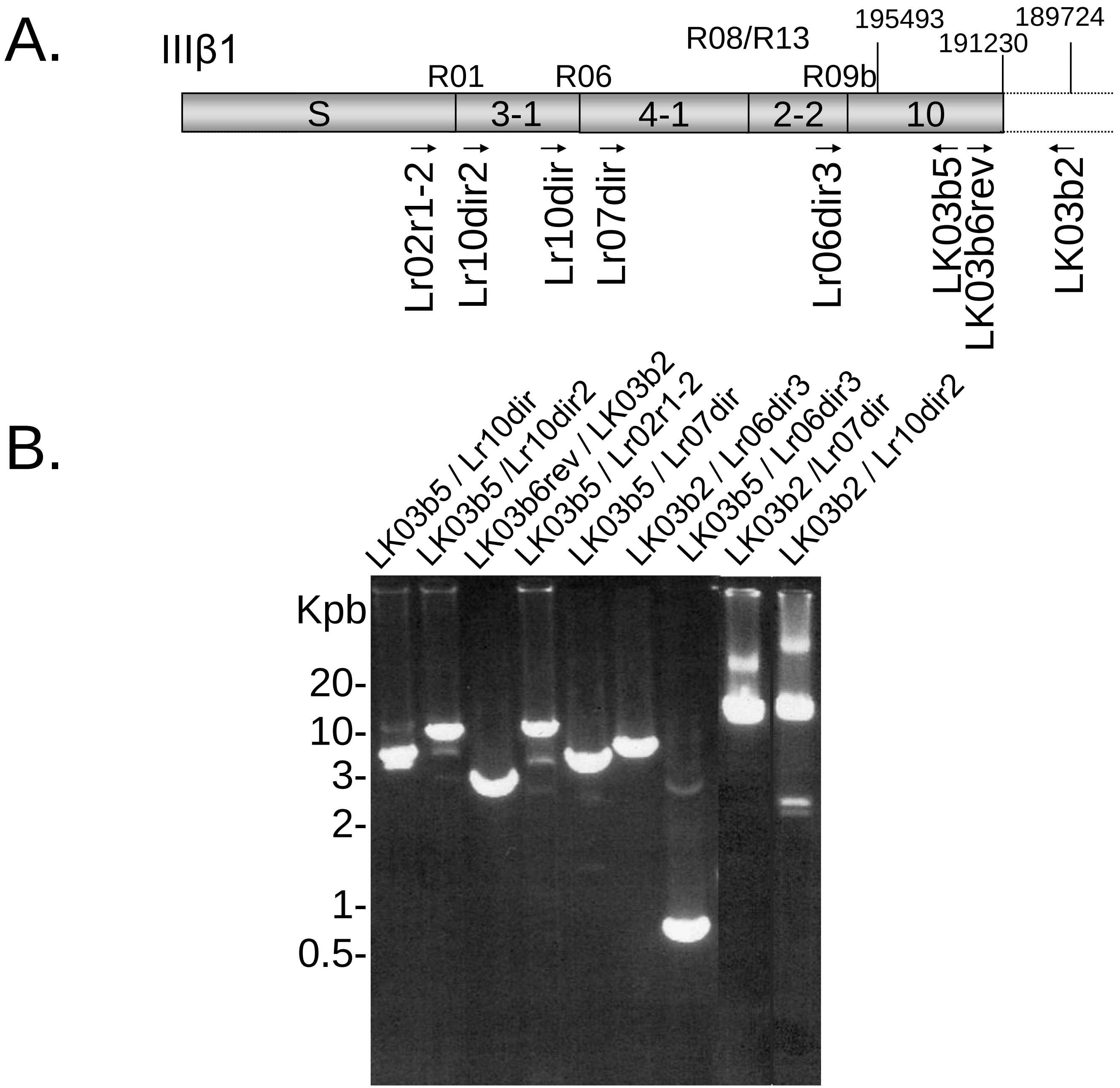

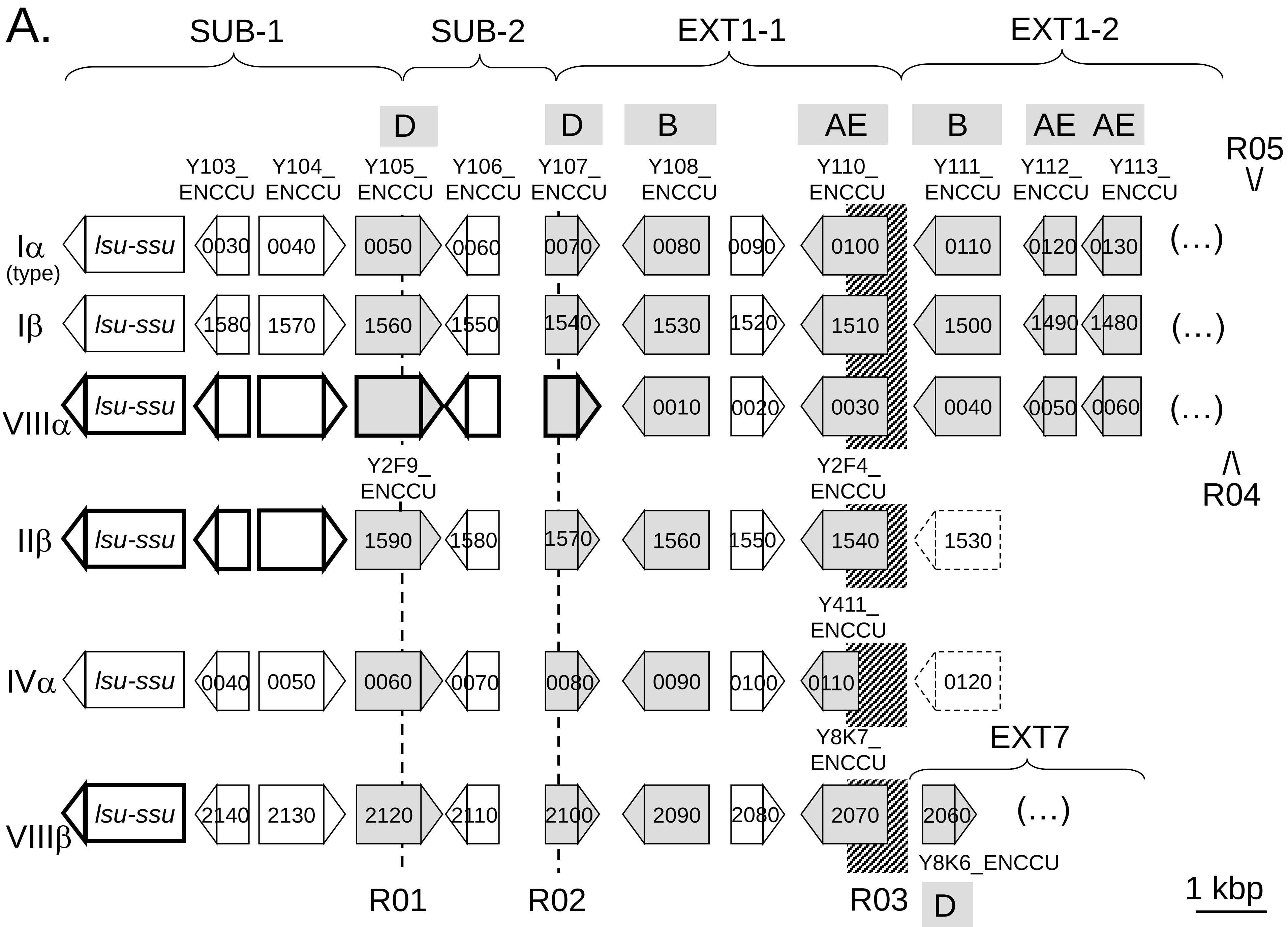

A.
SUB-1
SUB-2
EXT1-1
EXT1-2
D
D
B
AE
B
AE AE
Y103_ ENCCU
Y104_ ENCCU
Y105_ ENCCU
Y106_ ENCCU
Y107_ ENCCU
Y108_ ENCCU
Y110_ ENCCU
Y111_ ENCCU
Y112_ ENCCU
Y113_ ENCCU
R05
Iα (type)
lsu-ssu 0030 0040 0050 0060 0070 0080 0090 0100 0110 0120 0130 (…)
Iβ
lsu-ssu 1580 1570 1560 1550 1540 1530 1520 1510 1500 1490 1480 (…)
VIIIα
lsu-ssu 0010 0020 0030 0040 0050 0060 (…)
Y2F9_ ENCCU
Y2F4_ ENCCU
R04
IIβ
lsu-ssu 1590 1580 1570 1560 1550 1540 1530
Y411_ ENCCU
IVα
lsu-ssu 0040 0050 0060 0070 0080 0090 0100 0110 0120
Y8K7_ ENCCU
EXT7
VIIIβ
lsu-ssu 2140 2130 2120 2110 2100 2090 2080 2070 2060 (…)
Y8K6_ENCCU
R01
R02
R03
D
1 kbp

Supplementary Figure S5

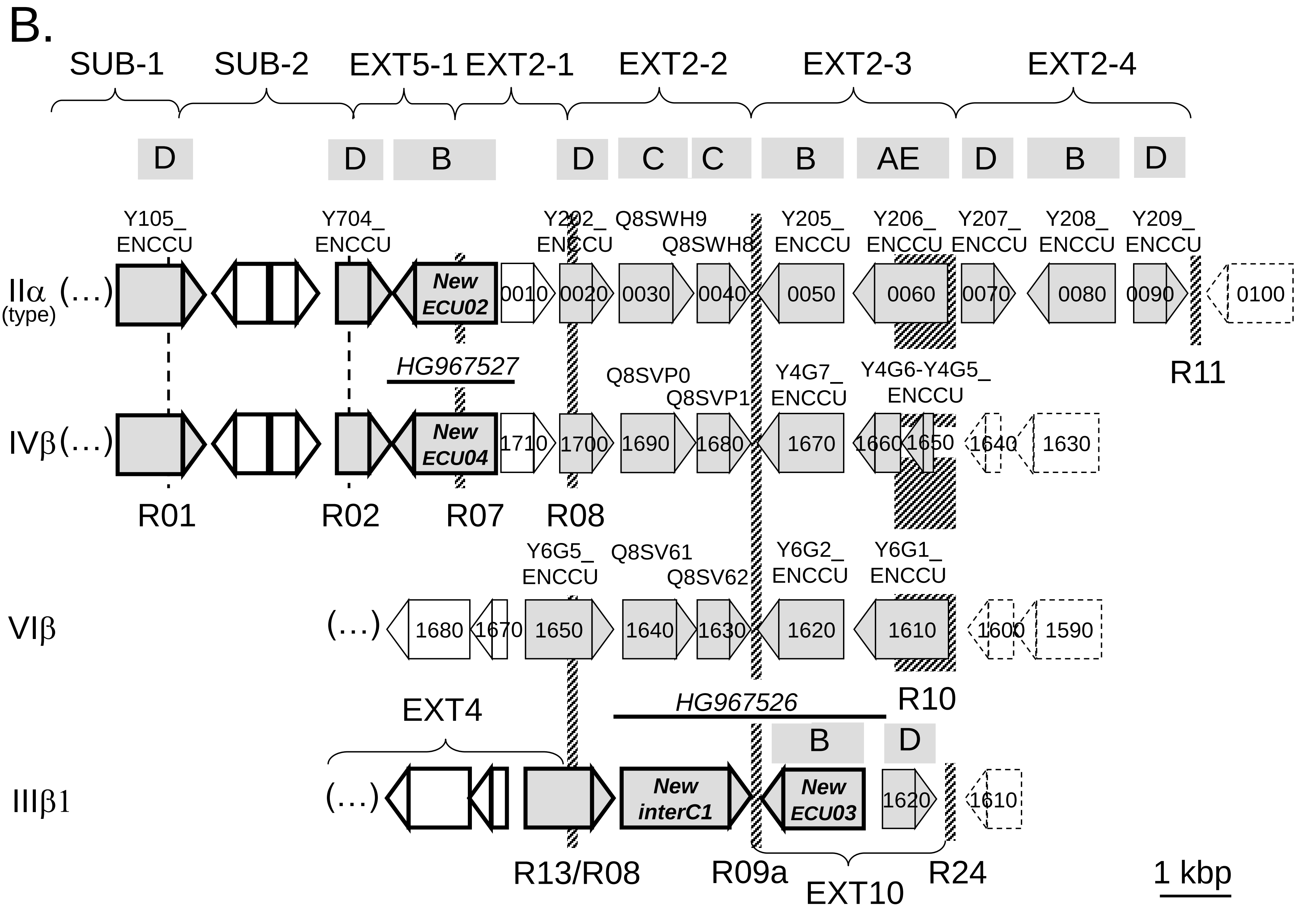

C.

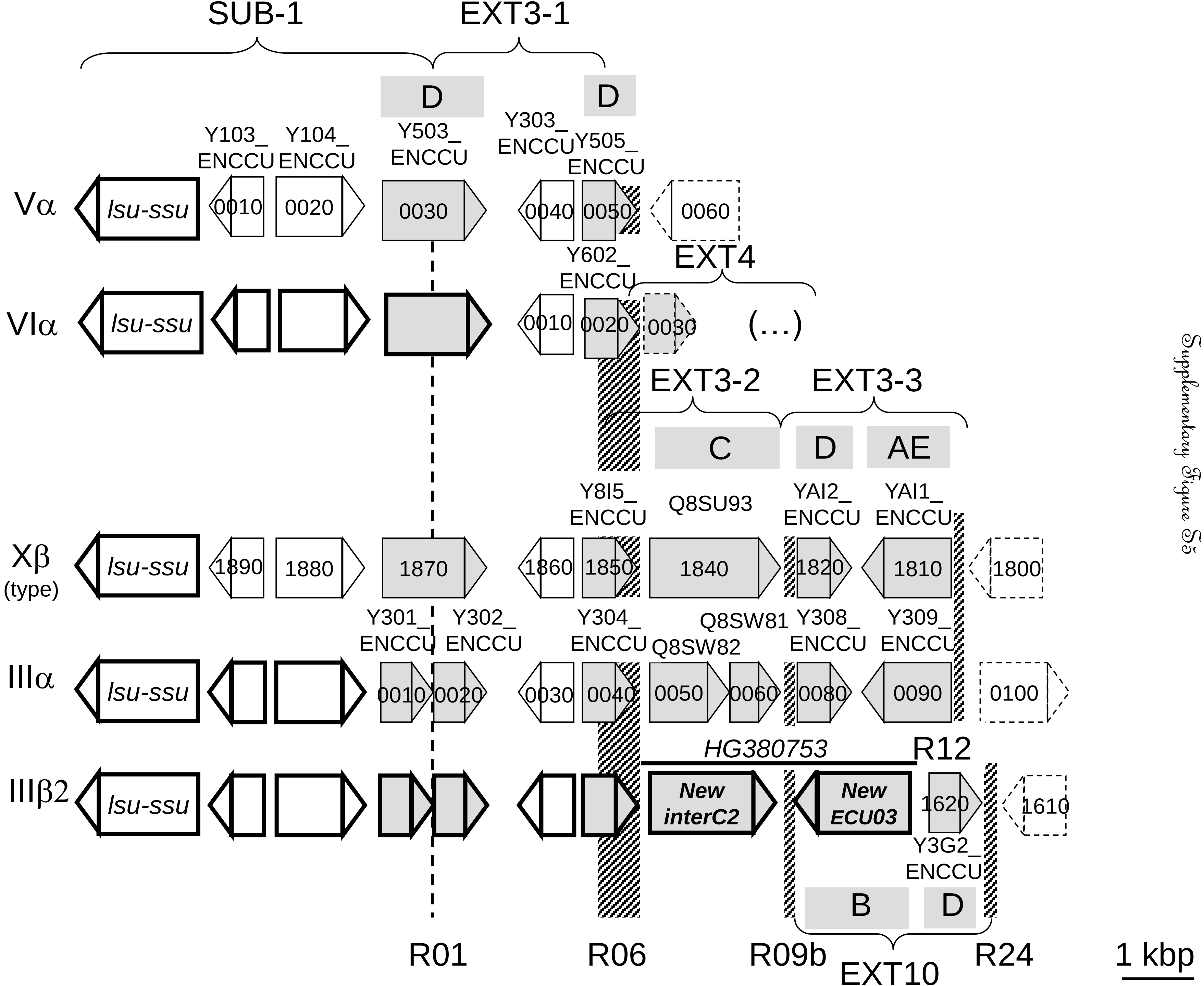

Supplementary Figure S5

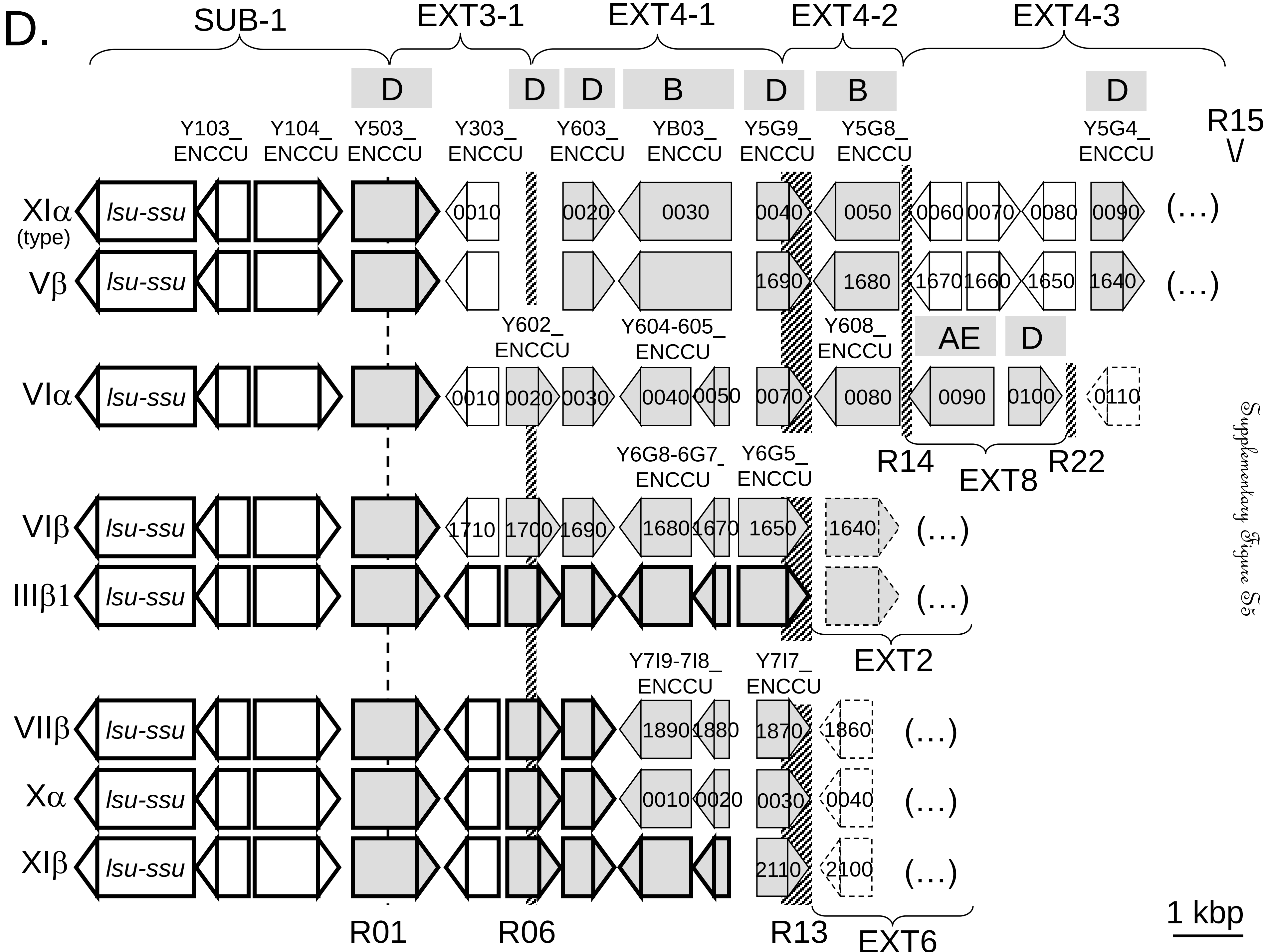

Supplementary Figure S5

E.

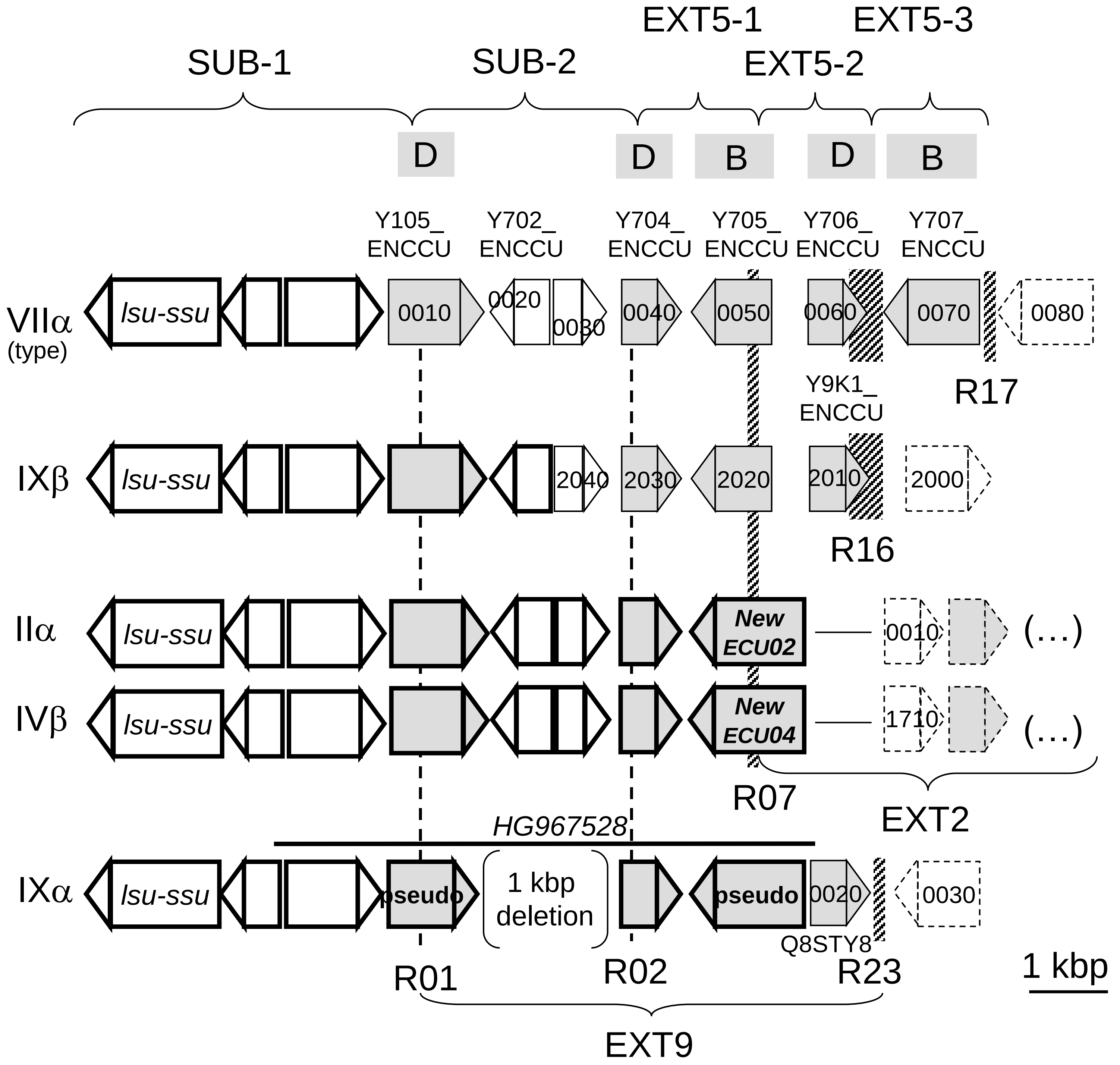

Supplementary Figure S5

F.

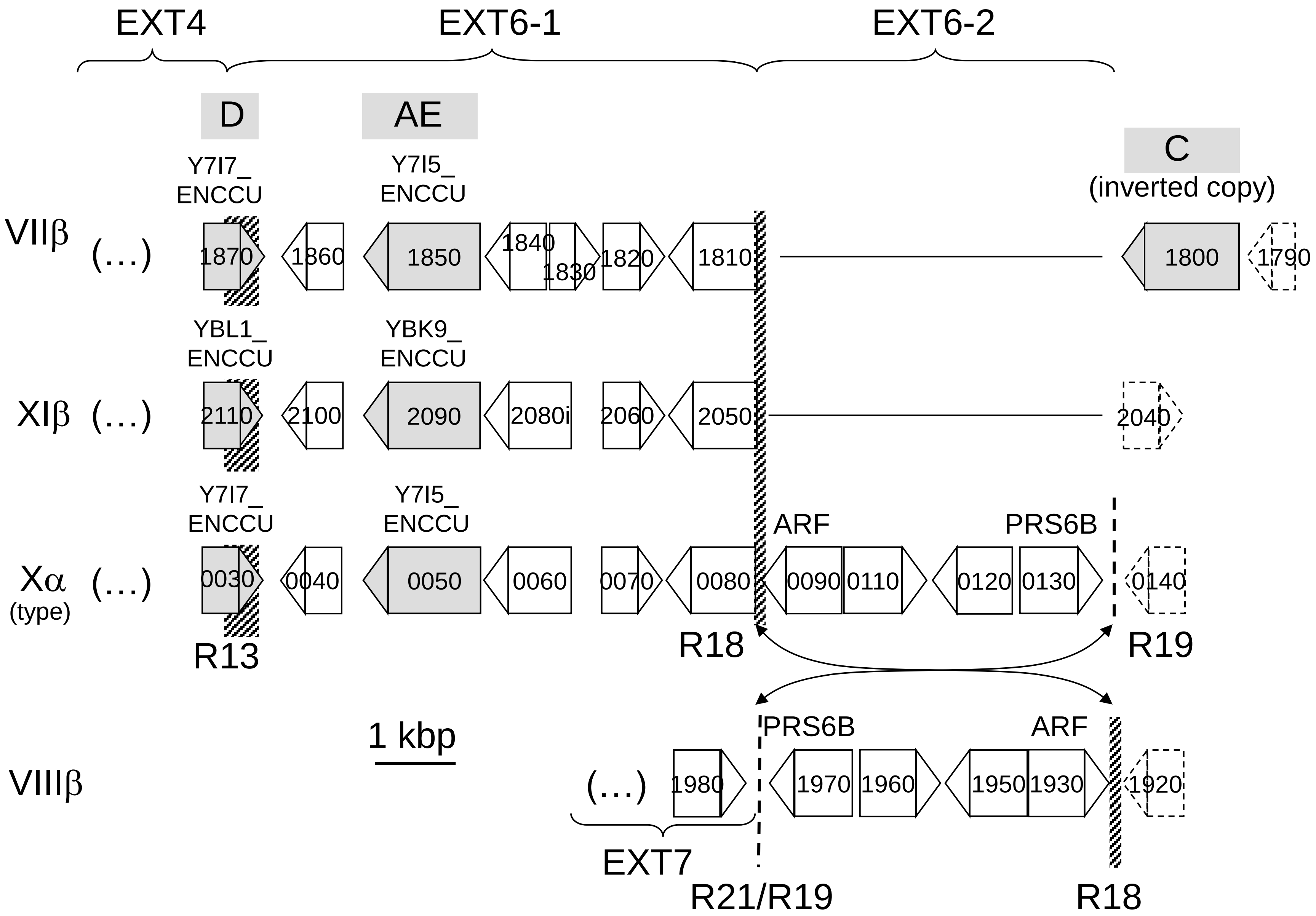
EXT4
EXT6-1
EXT6-2
D
AE
C
(inverted copy)
Y7I7_
ENCCU
Y7I5_
ENCCU
VIIβ
(…)
1870
1860
1850
1840
1830
1820
1810
1800
1790
YBL1_
ENCCU
YBK9_
ENCCU
XIβ
(…)
2110
2100
2090
2080i
2060
2050
2040
Y7I7_
ENCCU
Y7I5_
ENCCU
ARF
PRS6B
Xα
(type)
(…)
0030
0040
0050
0060
0070
0080
0090
0110
0120
0130
0140
R13
R18
R19
1 kbp
PRS6B
ARF
VIIIβ
(…)
1980
1970
1960
1950
1930
1920
EXT7
R21/R19
R18

G.

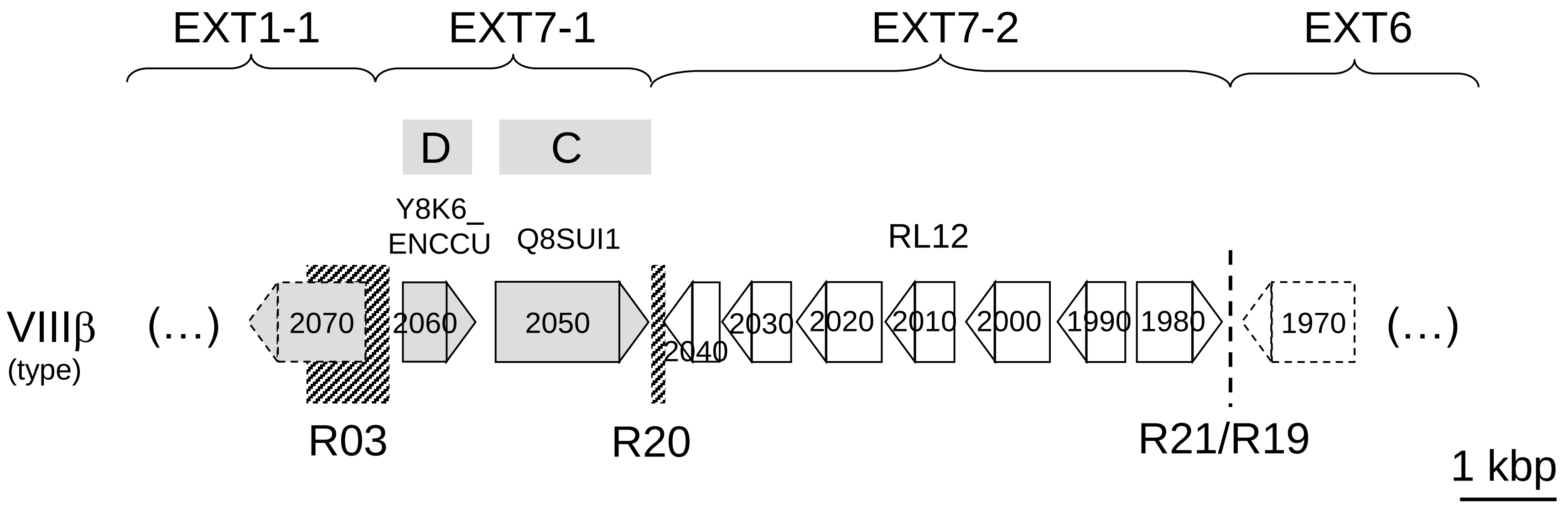
EXT1-1
EXT7-1
EXT7-2
EXT6
D
C
Y8K6_
ENCCU
Q8SUI1
RL12
VIIIβ
(type)
(…)
2070
2060
2050
2040
2030
2020
2010
2000
1990
1980
1970
(…)
R03
R20
R21/R19
1 kbp

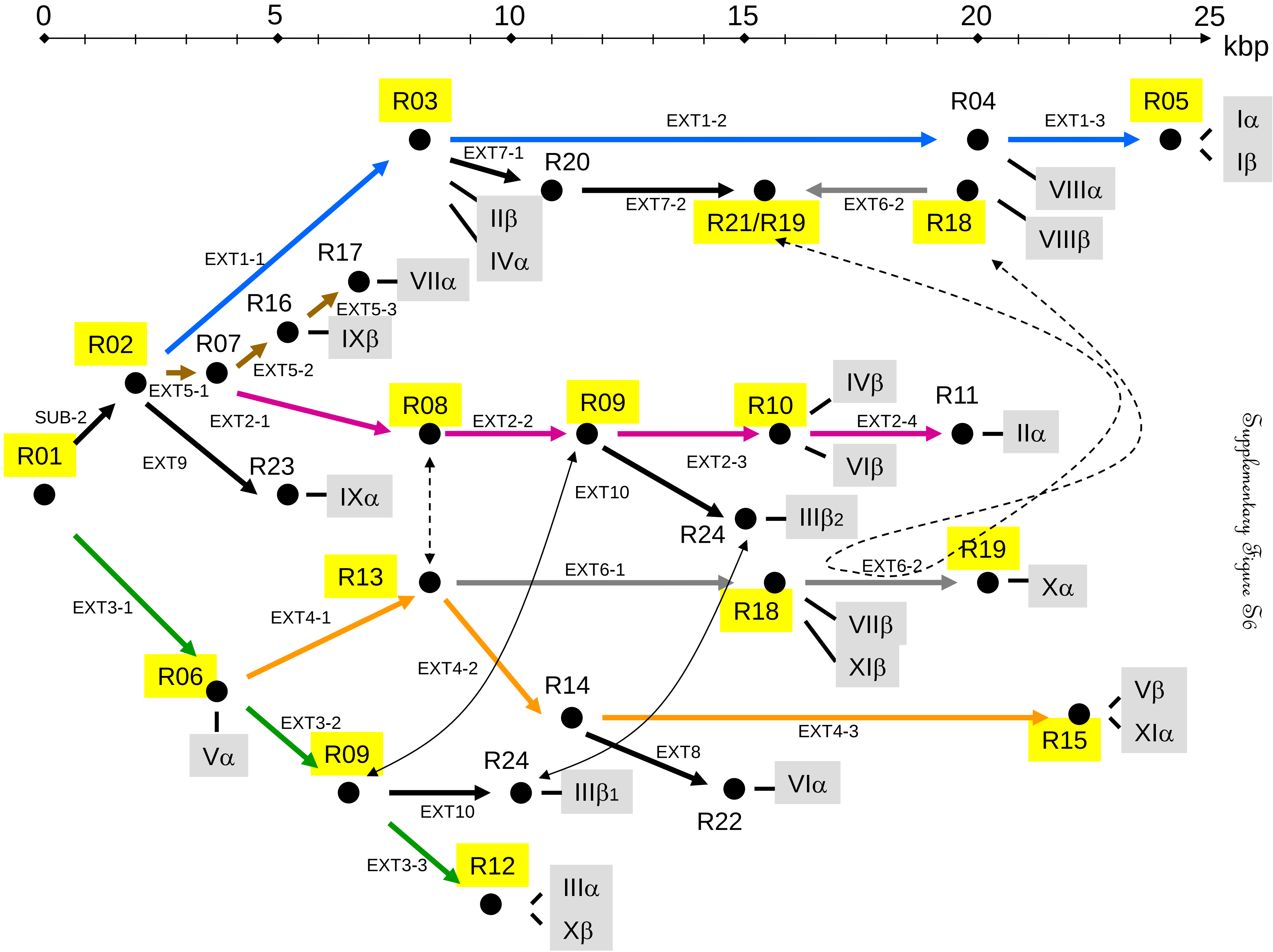

Supplementary Figure S6

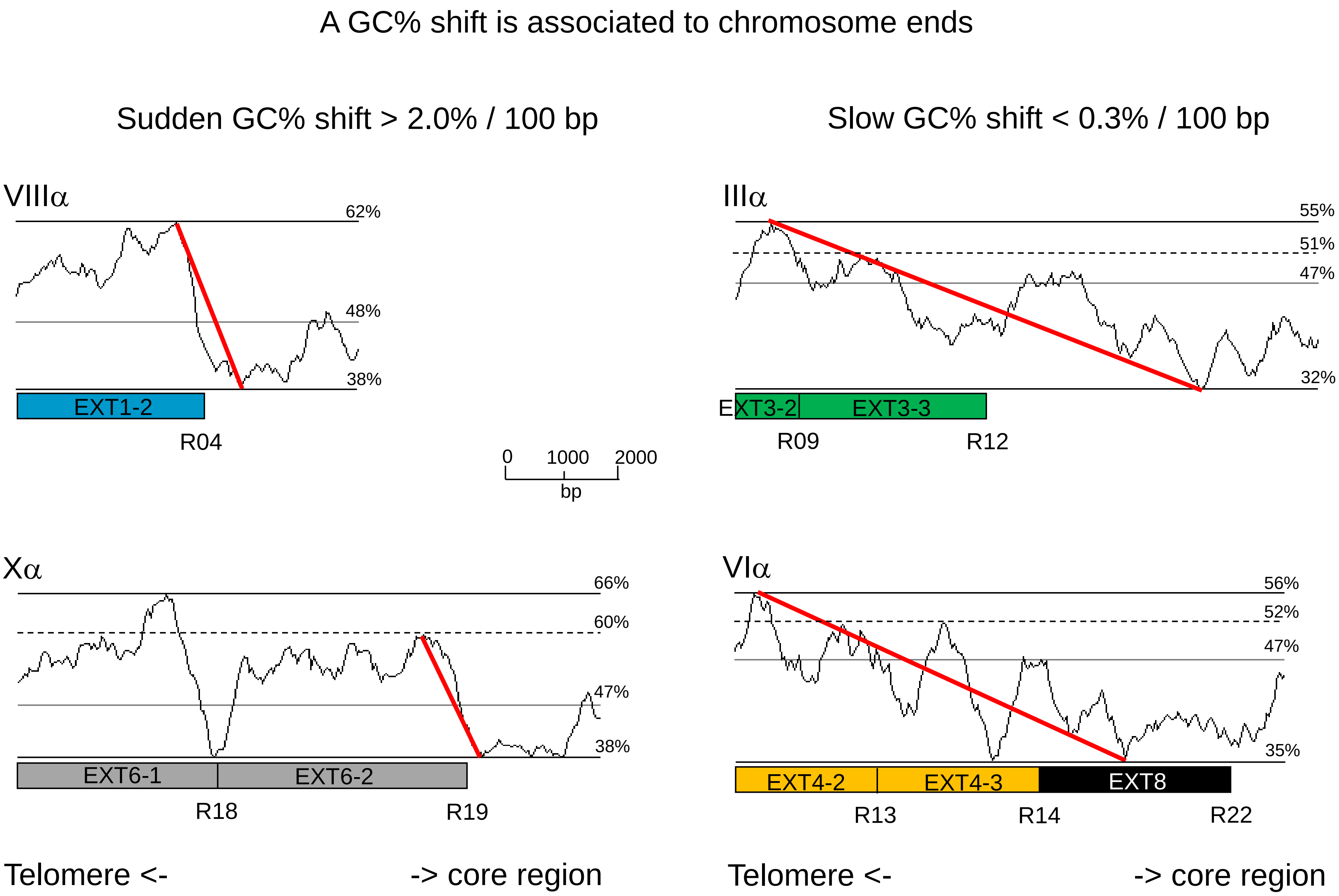

Supplementary Figure S7

Supplementary Figure S8

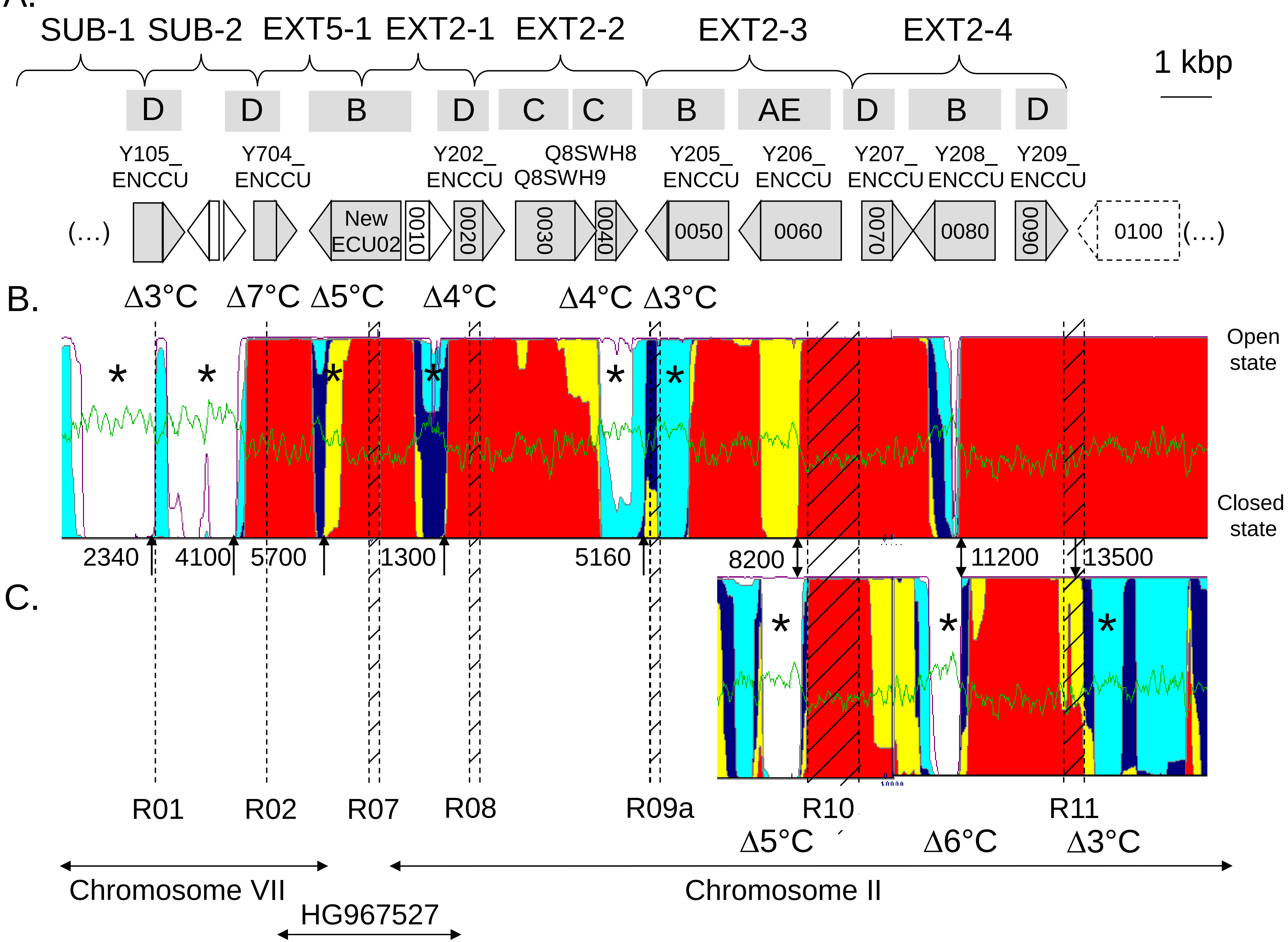

Supplementary Figure S9

InterAE/InterB

ECU04_1670 (586 aa),
Y4G7_ENCCU (Q8SVP2)

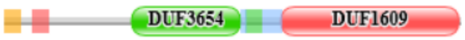

Source	Domain	Start	End
sig_p	n/a	1	21
transmembrane	n/a	37	56
low_complexity	n/a	164	180
Pfam A	DUF3654	165	304
disorder	n/a	168	182
low_complexity	n/a	219	233
disorder	n/a	276	280
low_complexity	n/a	305	321
coiled_coil	n/a	312	332
disorder	n/a	315	316
disorder	n/a	326	386
low_complexity	n/a	330	373
Pfam A	DUF1609	357	585
disorder	n/a	409	412
disorder	n/a	417	418
disorder	n/a	451	455
disorder	n/a	525	530

InterC

ECU10_1840 (584 aa),
Q8SU93_ENCCU

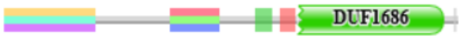

Source	Domain	Start	End
Pfam B	Pfam-B_453445	1	117
Pfam B	Pfam-B_2347	216	277
coiled_coil	n/a	325	345
transmembrane	n/a	357	374
Pfam A	DUF1686	380	566
transmembrane	n/a	386	405
transmembrane	n/a	425	442
transmembrane	n/a	449	468
transmembrane	n/a	474	492
transmembrane	n/a	504	527
low_complexity	n/a	510	524
transmembrane	n/a	539	563
disorder	n/a	582	583

InterD

ECU01_0070/ECU01_1540/ECU02_1570/
ECU04_0080/ECU08_2100 (254 aa),
Y107_ENCCU (Q8ST97)

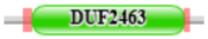

Source	Domain	Start	End
transmembrane	n/a	26	44
Pfam A	DUF2463	37	231
transmembrane	n/a	56	75
transmembrane	n/a	81	101
low_complexity	n/a	83	104
transmembrane	n/a	113	130
transmembrane	n/a	150	168
low_complexity	n/a	154	168
transmembrane	n/a	175	191
transmembrane	n/a	211	238

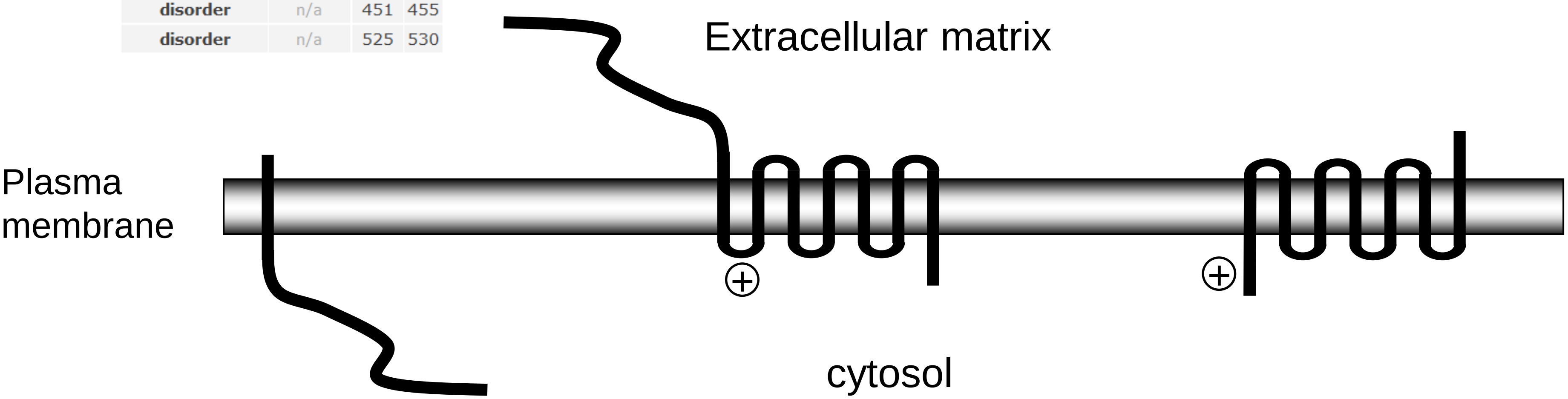

A.

Supplementary Figure S10

All Results	Ortholog Groups	Microsporidia																				
		M.Anncaliia (nr Genes: 0)		M.Edhazardia	M.Encephalitozoon (nr Genes: 38)				M.Encephalitozoon (nr Genes: 3)		M.Encephalitozoon	M.Encephalitozoon	M.Enterocytozoon	M.Mitosporidium	M.Nematocida (nr Genes: 0)		M.Nematocida (nr Genes: 0)		M.Nosema	M.Nosema	M.Ordospora	M.Spragu
		algerae PRA109	algerae PRA339	aedis USNM 41457	cuniculi EC1	cuniculi EC2	cuniculi EC3	cuniculi GB-M1	hellem ATCC 50504	hellem Swiss	intestinalis ATCC 50506	romaleae SJ-2008	bieneusi H348	daphniae UGP3	parisii ERTm1	parisii ERTm3	sp. 1 ERTm2	sp. 1 ERTm6	bombycis CQ1	ceranae BRL01	colligata OC4	lophii 42_110
66	3	0	0	0	7	8	8	36	3	1	3	0	0	0	0	0	0	0	0	0	0	0

interAE, *interB* genes (UPF0329)

Gene ID	Genomic Location
ECU01_0100	AL391737: 20,249 - 22,108 (-)
ECU01_0110	AL391737: 22,867 - 24,645 (-)
ECU01_0120	AL391737: 25,107 - 25,706 (-)
ECU01_1490	AL391737: 184,277 - 184,876 (+)
ECU01_1500	AL391737: 185,338 - 187,116 (+)
ECU01_1510	AL391737: 187,875 - 189,734 (+)
ECU02_0050	AL590442: 5,448 - 7,019 (-)
ECU02_0060	AL590442: 7,441 - 9,249 (-)
ECU02_0080	AL590442: 10,535 - 12,274 (-)
ECU02_1540	AL590442: 187,833 - 189,683 (+)
ECU03_0090	AL590443: 8,576 - 10,444 (-)
ECU04_0110	AL590444: 16,684 - 17,325 (-)
ECU04_1660	AL590444: 209,875 - 210,684 (+)
ECU04_1670	AL590444: 211,107 - 212,867 (+)
ECU05_1680	AL590445: 207,816 - 209,654 (+)
ECU06_0040	AL590446: 4,779 - 6,212 (-)
ECU06_0080	AL590446: 8,293 - 10,035 (-)
ECU06_0090	AL590446: 11,059 - 12,879 (-)
ECU06_1610	AL590446: 205,815 - 207,638 (+)
ECU06_1620	AL590446: 207,962 - 209,701 (+)
ECU06_1680	AL590446: 214,140 - 215,516 (+)
ECU07_0050	AL590447: 5,249 - 5,899 (-)
ECU07_0070	AL590447: 7,406 - 9,253 (-)
ECU07_1850	AL590447: 218,977 - 220,881 (+)
ECU07_1890	AL590447: 224,581 - 225,882 (+)
ECU08_0030	AL590448: 3,463 - 5,322 (-)
ECU08_0040	AL590448: 6,081 - 7,859 (-)
ECU08_0050	AL590448: 8,321 - 8,920 (-)
ECU08_2070	AL590448: 226,792 - 228,651 (+)
ECU09_2020	AL590451: 248,027 - 248,677 (+)
ECU10_0010	AL590449: 695 - 1,996 (-)
ECU10_0050	AL590449: 5,696 - 7,600 (-)
ECU10_1810	AL590449: 250,014 - 251,879 (+)
ECU11_0030	AL590450: 4,681 - 6,606 (-)
ECU11_0050	AL590450: 8,173 - 10,011 (-)
ECU11_2090	AL590450: 263,188 - 265,092 (+)

GB-M1 11/11 chromosomes

Sequence	Organism	Chromosome	#Genes	Length	Gene Locations
AL391737	*Encephalitozoon cuniculi GB-M1*	I	6	209982	
AL590442	*Encephalitozoon cuniculi GB-M1*	II	4	197426	
AL590443	*Encephalitozoon cuniculi GB-M1*	III	1	194439	
AL590444	*Encephalitozoon cuniculi GB-M1*	IV	3	218329	
AL590445	*Encephalitozoon cuniculi GB-M1*	V	1	211018	
AL590446	*Encephalitozoon cuniculi GB-M1*	VI	6	220294	
AL590447	*Encephalitozoon cuniculi GB-M1*	VII	4	226576	
AL590448	*Encephalitozoon cuniculi GB-M1*	VIII	4	238147	
AL590449	*Encephalitozoon cuniculi GB-M1*	X	3	262797	
AL590450	*Encephalitozoon cuniculi GB-M1*	XI	3	267509	
AL590451	*Encephalitozoon cuniculi GB-M1*	IX	1	251002	

EC1 4/11 chromosomes

Sequence	Organism	Chromosome	#Genes	Length	Gene Locations
ECI_CH02	*Encephalitozoon cuniculi EC1*	2	1	180123	
ECI_CH04	*Encephalitozoon cuniculi EC1*	4	1	192854	
ECI_CH06	*Encephalitozoon cuniculi EC1*	6	4	201713	
ECI_CH07	*Encephalitozoon cuniculi EC1*	7	1	207567	
Sequence	Organism	Chromosome	#Genes	Length	Gene Locations

EC3 5/11 chromosomes

Sequence	Organism	Chromosome	#Genes	Length	Gene Locations
ECIII_CH02	*Encephalitozoon cuniculi EC3*	2	1	177125	
ECIII_CH03	*Encephalitozoon cuniculi EC3*	3	1	184824	
ECIII_CH06	*Encephalitozoon cuniculi EC3*	6	4	201381	
ECIII_CH07	*Encephalitozoon cuniculi EC3*	7	1	211783	
ECIII_CH08	*Encephalitozoon cuniculi EC3*	8	1	217589	
Sequence	Organism	Chromosome	#Genes	Length	Gene Locations

B.

Supplementary Figure S10

All Results	Ortholog Groups	Microsporidia																				
		M.Anncaliia (nr Genes: 0)		*M.Edhazardia*	*M.Encephalitozoon* (nr Genes: 23)				*M.Encephalitozoon* (nr Genes: 7)		*M.Encephalitozoon*	*M.Encephalitozoon*	*M.Enterocytozoon*	*M.Mitosporidium*	*M.Nematocida* (nr Genes: 0)		*M.Nematocida* (nr Genes: 0)		*M.Nosema*	*M.Nosema*	*M.Ordospora*	*M.Spragu*
		algerae PRA109	algerae PRA339	aedis USNM 41457	cuniculi EC1	cuniculi EC2	cuniculi EC3	cuniculi GB-M1	hellem ATCC 50504	hellem Swiss	intestinalis ATCC 50506	romaleae SJ-2008	bieneusi H348	daphniae UGP3	parisii ERTm1	parisii ERTm3	sp. 1 ERTm2	sp. 1 ERTm6	bombycis CQ1	ceranae BRL01	colligata OC4	lophii 42_110
69	4	0	0	0	13	7	8	23	5	4	4	5	0	0	0	0	0	0	0	0	0	0

interC genes (UPF0329)

Gene ID	Genomic Location
ECU02_0030	AL590442: 3,287 - 4,384 (+)
ECU02_0040	AL590442: 4,524 - 4,895 (+)
ECU03_0050	AL590443: 5,400 - 6,665 (+)
ECU03_0060	AL590443: 6,782 - 7,153 (+)
ECU04_1680	AL590444: 213,230 - 213,829 (-)
ECU04_1690	AL590444: 213,718 - 214,836 (-)
ECU06_1630	AL590446: 210,069 - 210,440 (-)
ECU06_1640	AL590446: 210,557 - 211,744 (-)
ECU07_1800	AL590447: 213,403 - 215,382 (+)
ECU08_2050	AL590448: 223,792 - 225,192 (-)
ECU09_0530	AL590451: 68,590 - 70,473 (-)
ECU09_0540	AL590451: 70,894 - 72,597 (+)
ECU09_0580	AL590451: 75,755 - 77,383 (+)
ECU09_0590	AL590451: 77,832 - 79,427 (-)
ECU09_1120	AL590451: 130,649 - 132,373 (+)
ECU09_1160	AL590451: 133,647 - 135,251 (-)
ECU09_1530	AL590451: 174,234 - 175,946 (-)
ECU09_1550	AL590451: 178,654 - 180,429 (+)
ECU10_0450	AL590449: 50,646 - 52,472 (-)
ECU10_0460	AL590449: 52,845 - 53,708 (+)
ECU10_1840	AL590449: 253,302 - 255,056 (-)
ECU11_0250	AL590450: 33,269 - 34,918 (-)
ECU11_0290	AL590450: 40,082 - 41,680 (+)

GB-M1 9/11 chromosomes

Sequence	Organism	Chromosome	#Genes	Length	Gene Locations
AL590442	*Encephalitozoon cuniculi GB-M1*	II	2	197426	
AL590443	*Encephalitozoon cuniculi GB-M1*	III	2	194439	
AL590444	*Encephalitozoon cuniculi GB-M1*	IV	2	218329	
AL590446	*Encephalitozoon cuniculi GB-M1*	VI	2	220294	
AL590447	*Encephalitozoon cuniculi GB-M1*	VII	1	226576	
AL590448	*Encephalitozoon cuniculi GB-M1*	VIII	1	238147	
AL590449	*Encephalitozoon cuniculi GB-M1*	X	3	262797	
AL590450	*Encephalitozoon cuniculi GB-M1*	XI	2	267509	
AL590451	*Encephalitozoon cuniculi GB-M1*	IX	8	251002	

EC1 5/11 chromosomes

Sequence	Organism	Chromosome	#Genes	Length	Gene Locations
ECI_CH07	*Encephalitozoon cuniculi EC1*	7	1	207567	
ECI_CH08	*Encephalitozoon cuniculi EC1*	8	1	210542	
ECI_CH09a	*Encephalitozoon cuniculi EC1*	9a	5	133910	
ECI_CH09b	*Encephalitozoon cuniculi EC1*	9b	2	113009	
ECI_CH10	*Encephalitozoon cuniculi EC1*	10	2	235245	
ECI_CH11	*Encephalitozoon cuniculi EC1*	11	2	249484	

EC3 5/11 chromosomes

Sequence	Organism	Chromosome	#Genes	Length	Gene Locations
ECIII_CH07	*Encephalitozoon cuniculi EC3*	7	1	211783	
ECIII_CH08	*Encephalitozoon cuniculi EC3*	8	1	217589	
ECIII_CH09b	*Encephalitozoon cuniculi EC3*	9b	1	5946	
ECIII_CH09e	*Encephalitozoon cuniculi EC3*	9e	1	69589	
ECIII_CH10	*Encephalitozoon cuniculi EC3*	10	2	235461	
ECIII_CH11	*Encephalitozoon cuniculi EC3*	11	2	249260	

C.

Supplementary Figure S10

All Results	Ortholog Groups	Microsporidia																				
		M.Anncaliia (nr Genes: 0)		M.Edhazardia	M.Encephalitozoon (nr Genes: 50)				M.Encephalitozoon (nr Genes: 8)		M.Encephalitozoon	M.Encephalitozoon	M.Enterocytozoon	M.Mitosporidium	M.Nematocida (nr Genes: 0)		M.Nematocida (nr Genes: 0)		M.Nosema	M.Nosema	M.Ordospora	M.Spragu
		algerae PRA109	algerae PRA339	aedis USNM 41457	cuniculi EC1	cuniculi EC2	cuniculi EC3	cuniculi GB-M1	hellem ATCC 50504	hellem Swiss	intestinalis ATCC 50506	romaleae SJ-2008	bieneusi H348	daphniae UGP3	parisii ERTm1	parisii ERTm3	sp. 1 ERTm2	sp. 1 ERTm6	bombycis CQ1	ceranae BRL01	colligata OC4	lophii 42_110
108	8	0	0	0	12	14	12	49	4	7	4	6	0	0	0	0	0	0	0	0	0	0

interD genes (UPF0328)

Gene ID	Genomic Location
ECU01_0100	AL391737: 20,249 - 22,108 (-)
ECU01_0110	AL391737: 22,867 - 24,645 (-)
ECU01_0120	AL391737: 25,107 - 25,706 (-)
ECU01_1490	AL391737: 184,277 - 184,876 (+)
ECU01_1500	AL391737: 185,338 - 187,116 (+)
ECU01_1510	AL391737: 187,875 - 189,734 (+)
ECU02_0050	AL590442: 5,448 - 7,019 (-)
ECU02_0060	AL590442: 7,441 - 9,249 (-)
ECU02_0080	AL590442: 10,535 - 12,274 (-)
ECU02_1540	AL590442: 187,833 - 189,683 (+)
ECU03_0090	AL590443: 8,576 - 10,444 (-)
ECU04_0110	AL590444: 16,684 - 17,325 (-)
ECU04_1660	AL590444: 209,875 - 210,684 (+)
ECU04_1670	AL590444: 211,107 - 212,867 (+)
ECU05_1680	AL590445: 207,816 - 209,654 (+)
ECU06_0040	AL590446: 4,779 - 6,212 (-)
ECU06_0080	AL590446: 8,293 - 10,035 (-)
ECU06_0090	AL590446: 11,059 - 12,879 (-)
ECU06_1610	AL590446: 205,815 - 207,638 (+)
ECU06_1620	AL590446: 207,962 - 209,701 (+)
ECU06_1680	AL590446: 214,140 - 215,516 (+)
ECU07_0050	AL590447: 5,249 - 5,899 (-)
ECU07_0070	AL590447: 7,406 - 9,253 (-)
ECU07_1850	AL590447: 218,977 - 220,881 (+)
ECU07_1890	AL590447: 224,581 - 225,882 (+)
ECU08_0040	AL590448: 6,081 - 7,859 (-)
ECU08_0050	AL590448: 8,321 - 8,920 (-)
ECU08_2070	AL590448: 226,792 - 228,651 (+)
ECU09_2020	AL590451: 248,027 - 248,677 (+)
ECU10_0010	AL590449: 695 - 1,996 (-)
ECU10_0050	AL590449: 5,696 - 7,600 (-)
ECU10_1810	AL590449: 250,014 - 251,879 (+)
ECU11_0030	AL590450: 4,681 - 6,606 (-)
ECU11_0050	AL590450: 8,173 - 10,011 (-)
ECU11_2090	AL590450: 263,188 - 265,092 (+)

GB-M1 11/11 chromosomes

Sequence	Organism	Chromosome	#Genes	Length	Gene Locations
AL391737	*Encephalitozoon cuniculi GB-M1*	I	6	209982	
AL590442	*Encephalitozoon cuniculi GB-M1*	II	4	197426	
AL590443	*Encephalitozoon cuniculi GB-M1*	III	1	194439	
AL590444	*Encephalitozoon cuniculi GB-M1*	IV	3	218329	
AL590445	*Encephalitozoon cuniculi GB-M1*	V	1	211018	
AL590446	*Encephalitozoon cuniculi GB-M1*	VI	6	220294	
AL590447	*Encephalitozoon cuniculi GB-M1*	VII	4	226576	
AL590448	*Encephalitozoon cuniculi GB-M1*	VIII	4	238147	
AL590449	*Encephalitozoon cuniculi GB-M1*	X	3	262797	
AL590450	*Encephalitozoon cuniculi GB-M1*	XI	3	267509	
AL590451	*Encephalitozoon cuniculi GB-M1*	IX	1	251002	
Sequence	Organism	Chromosome	#Genes	Length	Gene Locations

EC1 4/11 chromosomes

Sequence	Organism	Chromosome	#Genes	Length	Gene Locations
ECI_CH02	*Encephalitozoon cuniculi EC1*	2	1	180123	
ECI_CH04	*Encephalitozoon cuniculi EC1*	4	1	192854	
ECI_CH06	*Encephalitozoon cuniculi EC1*	6	4	201713	
ECI_CH07	*Encephalitozoon cuniculi EC1*	7	1	207567	
Sequence	Organism	Chromosome	#Genes	Length	Gene Locations

EC3 5/11 chromosomes

Sequence	Organism	Chromosome	#Genes	Length	Gene Locations
ECIII_CH02	*Encephalitozoon cuniculi EC3*	2	1	177125	
ECIII_CH03	*Encephalitozoon cuniculi EC3*	3	1	184824	
ECIII_CH06	*Encephalitozoon cuniculi EC3*	6	4	201381	
ECIII_CH07	*Encephalitozoon cuniculi EC3*	7	1	211783	
ECIII_CH08	*Encephalitozoon cuniculi EC3*	8	1	217589	
Sequence	Organism	Chromosome	#Genes	Length	Gene Locations
