# Supplementary material for: Subtelomere organization in the genome of the microsporidian Encephalitozoon cuniculi: patterns of repeated sequences and physicochemical signatures
Source: BMC Genomics. 2016 Jan 7;17:34. doi: 10.1186/s12864-015-1920-7 (PMC4704409; doi:10.1186/s12864-015-1920-7)
Supplement: Additional file 2: Table S1. — Primer list used for the determination of chromosome ends structure in Encephalitozoon cuniculi. (DOC 85 kb) [file 12864_2015_1920_MOESM2_ESM.doc]

Additional file 2: Table S1. Primer list used for the determination of chromosome ends structure in *Encephalitozoon cuniculi*.

| Primer | 5' to 3' oligonucleotides sequence | F/R° | Chromosome Region |
| --- | --- | --- | --- |
| LK01a | CAGGGCTCTTCTCTCAACCATTCCCTGTGG | R | Coding core |
| LK01b | CATTGCGTTCAGACGGACGGGAGTGGGAGC | R | Coding core |
| LK02a | ATGCAGGATGGCTACGAGTCTGTGACGCCG | R | Coding core |
| LK02b | TCACACTGCGATAGAGACGGGTCTTGACGG | R | Coding core |
| LK03a | CGGAGGGACTGCATTTCATGAGACCACGGC | R | Coding core |
| LK03b | CTGTGATGCCAGTGTCTGTGAATGAGGCGG | R | Coding core |
| LK03b2 | GAGTTGATGAGATTGTATTTAGGGATGACACTGG | R | Coding core |
| LK03b5 | GGAGTGCAGGAGTCCCTGAGAGTAAGGAAGAG | R | EXT10* |
| LK03b6rev | TCCGAGGAGCAGTGCATCAATCACAGTAA | F | EXT10* |
| LK04a | GGTTCTGGTCGACGCCATTGTCAACTCCGG | R | Coding core |
| LK04b | CTGCATAGTCGTTGGAGGGCGATAGGGCGC | R | Coding core |
| LK05a | AGCTTTGATTGCAGATCAGGCACACGCCCG | R | Coding core |
| LK05b | GAATGTCGAGTGATAGGGGAGGAATCCCGG | R | Coding core |
| LK06a | AGTATCCAGGTGTTGAGTCGAGTACCGGGC | R | Coding core |
| LK06b | TTTGAGGTCATTGTCTCTGAGTCGCCGGCC | R | Coding core |
| LK07a | ACCTCCTCAATGCTCAGGTTCGGGTCGGGC | R | Coding core |
| LK07b | CAACATTCTTTGGGTCGCAGACTAGCGCCG | R | Coding core |
| LK08a | TGGCAAGCGGAAGACAGGCAGGAAGCGTCG | R | Coding core |
| LK08b1 | ACCTCGACAGAATGAGCGATGAAGGCCGGC | R | Coding core |
| LK08b2 | CGACGGCAAGTGTCCGAAGGAAGTTACGCG | R | Coding core |
| LK09a | GCCTTGAGAAGGCAATAGCAGGAAGCCGCG | R | Coding core |
| Lk09a2 | GAGGCTAATGATGCTGAGAAGATAGACTC | R | EXT9 |
| Lk09a3 | CATAGTGGATGTCGTTTCGCTCCCAAACC | R | EXT9 |
| LK09b | AGTAGGTCCTGGTGCGATGCTTCTGCCACG | R | Coding core |
| LK10a | CTGAGGTAGTTCGGAATGTGGACAAGCGCC | R | Coding core |
| LK10b | TAAAGAAGGCAGTGGTGGTCGGGAATGCGC | R | Coding core |
| LK11a | ATGGAGGTAGGACATCTCATCCACAGGCGG | R | Coding core |
| LK11b | GCAGGCTACAGAAAATATATACTTGAGATTCT | R | Coding core |
| Lr01-1 | CATCCTTCAGAGCCGGTAAGTTGCCCCGCG | F | SUB |
| Lr01-2 | CACCATAGACACTATTGGAGCAGGTATTACCGCGGC | F | SUB |
| Lr02debut | CTCCTTGTCCAATCAGACAGAAGGTCCCACAACTCC | F | SUB |
| Lr02fin | CCGAAGCATCTGAATAACGTCAAGCAATACTTCGTCCAC | F | EXT1 |
| Lr02r1_1 | TTTCTGCTGCATTCGATGAACGATGAACTAAGCCGG | F | SUB |
| Lr02r1-2 | ACAACCACCGTCAGTCGCTGCCACTAGCCG | F | SUB |
| Lr02r1rev | GGAGTCCACGCAGTCTTATGAGGGGAGAGGGCC | R | SUB |
| Lr02r2 | TCTGGATAGACAGCACTGCACCGCCTGCCC | F | SUB |
| Lr02r3 | CTGCCCACCTGCTATCCACCTCCTGCGCCC | F | EXT1 |
| Lr02r3rev | AAGGGCGCAGGAGGTGGATAGCAGGTGGGC | R | EXT1 |
| Lr03fin | AGCCCTCCAAGCAGCCCAAGAACACAAGCCGCC | F | EXT1 |
| Lr03finrev | TGGCGGCTTGTGTTCTTGGGCTGCTTGGAGGGC | R | EXT1 |
| Lr03rev | AGGCAGAAGGTTGGGGAGAAATAGGGGATGCCGGCG | R | EXT1 |
| Lr05debrev | GCGCACAGGAGGAGGTGGCTAGGGGGC | R | EXT2 |
| Lr05debut | CCTCCTCCTGTGCGCTCCTCGTGCACAGCC | F | EXT2 |
| Lr05dir1 | CCGTTTTACTAGACTTCCAGGGAAATGTCT | F | EXT2* |
| Lr05dir2 | GGAGATGCAGAGTCATTGAAAGCACAGTAC | F | EXT2 |
| Lr05dir3 | TTGTCCCGGGACAGACTGGATTCACAGACACC | F | EXT2 |
| Lr06dir3 | CTACTGTGGGAGGAGGGAGGAAGAGTCCCAG | F | EXT2 |
| Lr07dir | GCACCACACCACATAAGGCAATGAGAACCCCGCC | F | EXT4 |
| Lr07rev | GGGAGCAATTTCGCCGTTTGGGAAGTGTGATGATGTGGG | R | EXT4 |
| Lr08dir | CACCGTGCTCTTCCAATCCTCATACTCAGCAGCCCAG | F | EXT4 |
| Lr08rev | GATACTGGGCTGCTGAGTATGAGGATTGGAAGAGCACGG | R | EXT4 |
| Lr10dir | CTGATCTGCCCAAACGCTCATTCACCACGTCCGG | F | EXT4 |
| Lr10dir2 | CCAATCCTATCACATGAAATCCATTTCATCGT | F | EXT3 |
| Lr11dir | TATACACACGAAAGGGGCCCGGGCCTGCCG | F | EXT3 |
| Lr11rev | CCATGCTTGGGCAGGCCCGGGCCCC | R | EXT3 |
| Lr12dir1 | TCCGAAGCAGCTCCTCTGCATCCCTGGCCC | F | EXT4 |
| Lr12dir2 | CCGCACAAGCACGCCAGCCCCCGCC | F | EXT4 |
| Lr12rev1 | GCTGCTTCGGATGGAGGAGAGTGGAAAGGTTCTAAGGC | R | EXT4 |
| Lr12rev2 | CGGGGGCTGGCGTGCTTGTGCGGGC | R | EXT4 |
| Lr13dir1 | CCTGAGGGCACATTGCACAAGGCACTCTAC | F | EXT5 |
| Lr13dir2 | GGTTGTTGATAATGCAATGATGTTATTCCCT | F | EXT5 |
| Lr13dir3 | AAGAGTCCTTTCCTCAAATTCATTATAC | F | EXT5 |
| Lr13dir4 | GCATCCATGAAGAACTTGTTCGCATCTGG | F | EXT5 |
| Lr13rev1 | CTTTCCCATTTATCGAGGGTGAATGCAGTGA | R | EXT5 |
| Lr13rev2 | ATCGTATCAGGATGACTACGGGAAATAGGA | R | EXT5 |
| Lr14dir | GCCACTCCCACAAGCCACACTGCCCGCC | F | EXT2* |
| Lr14rev | GGCGGGCAGTGTGGCTTGTGGGAGTGCC | R | EXT2* |
| Lr15dir | GCGCGCGGATACTTCCTCGGGACGGG | F | EXT1 |
| Lr15rev | CCCGTCCCGAGGAAGTATCCCGCGCGC | R | EXT1 |
| rDNAFw1 | CTGGGGCAGTAGGGAGCTCTTTTCG | F | SUB |
| rDNAFw2 | GGAAGCGAAGGCTGTGCTCTTGGAC | F | SUB |
| rDNARv1 | CACAGACAGGGCTCAGGAGAGGTTC | R | SUB |
| rDNARv2 | GGTGATCCTCTGTCAACGCACGGGG | R | SUB |
|  |  |  |  |

* Specific of one chromosome, ° F/R: primer orientation, F: Forward, R: revers (Additional file 1: Figure S3A)
